# Role of NK cells in immune escape in patients with classical paroxysmal nocturnal haemoglobinuria

**DOI:** 10.1002/ctm2.70542

**Published:** 2025-12-17

**Authors:** Chaomeng Wang, Yan Yang, Wei Wang, Liyan Li, Mengting Che, Yingying Chen, Honglei Wang, Zhaoyun Liu, Lijuan Li, Hui Liu, Rong Fu

**Affiliations:** ^1^ Department of Hematology Tianjin Medical University General Hospital Tianjin People's Republic of China; ^2^ Tianjin Key Laboratory of Bone Marrow Failure and Malignant Hemopoietic Clone Control Tianjin People's Republic of China; ^3^ Tianjin Institute of Hematology Tianjin People's Republic of China; ^4^ State Key Laboratory of Experimental Hematology Tianjin People's Republic of China; ^5^ Shanghai Institute for Advanced Immunochemical Studies and School of Life Science and Technology ShanghaiTech University Shanghai People's Republic of China

**Keywords:** immune escape, natural killer cells, paroxysmal nocturnal haemoglobinuria

## Abstract

**Background:**

Paroxysmal nocturnal haemoglobinuria (PNH) is an acquired clonal haematopoietic stem cell disorder. Immune escape is crucial in PNH, and our previous studies revealed that natural killer (NK) cells potential participate in the immune escape of PNH. This study aimed to investigate the subtypes and functional changes of NK cells in PNH patients.

**Methods:**

We analysed CD59^+^ and CD59^−^ bone marrow mononuclear cells using single‐cell RNA sequencing (scRNA‐seq). The results were validated through flow cytometry and co‐culture experiments.

**Results:**

We classified NK cells into seven subtypes by scRNA‐seq, and found significant differences in the distribution of subtypes in CD59^+^ and CD59^−^ NK cell of PNH patients. Compared with controls, the proportion of active and adaptive NK cells was higher in CD59^+^ NK cells. Conversely, the proportion of CD56^bright^ NK cells and terminal NK cells was elevated in CD59^−^ NK cells. Additionally, the proportion of mature NK cells decreased in both the CD59^+^ and CD59^−^ groups. Gene ontology and Kyoto Encyclopedia of Genes and Genomes analysis revealed impaired function of CD59^−^ NK cells, whereas CD59^+^ NK cells showed minimal change. Furthermore, similar results were verified by flow cytometry and co‐culture in vivo and in vitro. And the proportion of NK cells was closely related to the proportion of CD8^+^ T cells and the clinical indicators of disease.

**Conclusions:**

The quantity and function of NK cells in PNH patients are insufficient, in which CD59^−^ NK cells have functional defects, whereas CD59^+^ NK cells were mainly activated and potential involved in immune escape by regulation of T cells.

## Graphical abstract

1. Functional impairment of CD59^‒^ NK cells in PNH is observed, with minimal change in CD59^+^ NK cells.

2. Direct killing ability of NK cells against target cells is reduced.

3. NK cells weakened T‐cell regulation, leading to hyperfunction of T lymphocytes and contributing to immune escape.

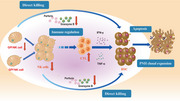

## 1 BACKGROUND

Paroxysmal nocturnal haemoglobinuria (PNH) is a rare clonal disease caused by a phosphatidylinositol glycan class A (*PIGA*) gene mutation in haematopoietic stem cells (HSCs), resulting in incomplete glycosyl‐phosphatidylinositol (GPI) anchored proteins (GPI‐APs) on the cell surface and presenting as intravascular haemolysis, bone marrow (BM) failure and thrombosis.^1^, ^2^ However, the mechanism by which the PNH clone, arising from a mutated HSC, expands in the BM remains unclear. Although current complement inhibitors effectively control haemolytic attacks in patients with PNH and improve their quality of life,^3^, ^4^ they do not eliminate PNH clones or cure the disease. Therefore, it is essential to study PNH clone formation and development. Current research indicates that immune escape, anti‐apoptotic mechanisms and secondary gene mutations contribute to PNH clone proliferation and immune factors play crucial roles in the occurrence and development of PNH.^5^

Natural killer (NK) cells are essential innate lymphocytes that modulate immune responses and contribute to cancer immunosurveillance.^6^ NK cells secrete cytokines and chemokines and release cytotoxic granules to exert their cytotoxic and immunoregulatory functions.^6^ Many recent studies have demonstrated NK cells participate in the pathogenesis of tumour and autoimmune diseases. The tumour microenvironment is highly complex, and immune escape is now recognised as a major factor in the development of tumour.^7^, ^8^, ^9^, ^10^ Some research also indicates that NK cell dysfunction is associated with haematological diseases. Studies have shown that the insufficient immune regulatory function of NK cells may be involved in the pathogenesis of aplastic anaemia,^11^ including a decrease in the number of NK cells^12^ and defects in function.^13^, ^14^, ^15^ The research also indicates that in patients with myelodysplastic syndrome, NK cells frequently display functional disorders, which are manifested as a reduction in cell numbers, alterations in antigen expression and a decrease in functional capacity.^16^, ^17^, ^18^ However, research on NK cells in PNH remains limited. Initial studies reported a decreased number of NK cells in the peripheral blood of patients with PNH,^19^ along with deficient NK cell activity.^20^ GPI‐APs play a significant role in cell signal transduction, cell adhesion, antigen presentation and membrane‐associated enzyme activities. Cells lacking the GPI‐APs ULBP1 and ULBP2 are less able to properly activate NK cells. Therefore, NK cells are more effective at killing GPI^+^ cells than GPI^−^ cells.^21^, ^22^ Immune cells lacking GPI‐APs may also affect immune recognition. PNH patients are mosaic with both PNH clone cells and healthy cells. However, it remains unclear how NK cell function changes in PNH and whether GPI‐AP absence affects their distribution and functions.

To explore these questions, we submitted CD59^+^ and CD59^−^ bone marrow mononuclear cells (BMMNCs) for single‐cell RNA sequencing (scRNA‐seq) analysis from patients with PNH and healthy controls (HCs).^23^ CD59^−^ BMMNCs represent GPI‐deficient PNH clones; CD59^+^ BMMNCs represent residual normal haematopoiesis. Similarly, CD59^+^ NK cells represent normal‐function NK cells, while CD59^−^ NK cells represent GPI‐deficient PNH clones. Our previous research suggests that activated normal T and NK cells may participate in the immune escape of PNH.^23^ Therefore, this study aimed to explore the role of NK cells, normal and abnormal cloned NK cells in classical PNH.

## 2 METHODS

### 2.1 Ethics approval statement

This study was approved by the Ethics Committee of Tianjin Medical University General Hospital (ethics approval no. IRB2025‐YX‐154‐01). Animal experiments were approved by the Animal Ethics Committee of the Tianjin Medical University General Hospital (ethical no. IRB2025‐DW‐35).

### 2.2 Patients

The scRNA‐seq information of four patients has been previously reported.^23^ In total, 26 patients (Table S1) with classical PNH and 27 HCs from the Department of Hematology, General Hospital of Tianjin Medical University, were enrolled between January 2022 and January 2024 to detect the quantity and function of NK cells. All patients were diagnosed according to the guidelines of the International PNH Research Group.^24^ Patients’ clinical information is presented in Table S1. In this study, BM and peripheral blood specimens were isolated from patients with classical PNH before treatment. CD59^+^ and CD59^‒^ cells representing normal and GPI‐deficient haematopoietic clones, respectively. Control cells were obtained from healthy individuals without PNH. All participants provided informed consent.

### 2.3 scRNA‐seq

We sorted CD59^+^ and CD59^−^ BMMNCs from four patients with classical PNH and normal BMMNCs from four HCs using flow cytometry and performed scRNA‐seq (Novogene Co. Ltd.). CD59^+^ NK cells, CD59^−^ NK cells and normal NK cells were analysed using scRNA‐seq.

### 2.4 Flow cytometry

PBMCs/BMMNCs were isolated from fresh samples via density gradient centrifugation and incubated for 30 min at 4°C with antibody cocktails in 100 µL phosphate‐buffered saline The antibody incubation protocols for NK cells and their subpopulations is included in the Supporting Information. The experiment was performed using the CytExpert flow cytometer, with dead cells and debris excluded through 4,6‐diamidino‐2‐phenylindole (DAPI) staining. Data were analysed using CytExpert software.

### 2.5 Cell culture and co‐culture

Primary human NK cells from PNH patients and HCs were isolated using negative magnetic bead selection (Miltenyi Biotec or Stemcell Technologies) and cultured in NK cell medium, which was added with 200 U/mL recombinant human interleukin (IL)‐2 (Roche) at 37°C in 5% CO_2_.

We employed CRISPR/Cas9 technology to establish a *PIG‐A* gene knockout K562 cell line (K562‐KO) as PNH cell model, which was verified to have a deficiency in CD59 expression by flow cytometry (Figure 7C). K562‐KO cells were cultured in Iscove's Modified Dulbecco's Medium (IMDM) with 1% penicillin‒streptomycin and 15% foetal bovine serum. NK cells were co‐cultured with K562‐KO cells at a 1:1 ratio for 12, 24 and 48 h.

### 2.6 Apoptosis analysis

To analyse NK cell function, the apoptosis of K562‐KO cells was measured using an Annexin V/APC apoptosis detection kit (BioLegend), following the manufacturer's instructions.

### 2.7 Enzyme‐linked immunosorbent assay

Supernatant interferon‐γ (IFN‐γ) levels from the co‐culture system were measured using an enzyme‐linked immunosorbent assay (ELISA) kit (EH0164, FineTest) following the manufacturer's instructions.

### 2.8 Mice

Flox and *Pig‐a* gene conditional knoncout (CKO) homozygous mice were generated^16^ and maintained by Cyagen Biosciences Inc. Peripheral blood from five flox mice and five CKO homozygous mice was collected via enucleation to assess NK cell quantity and function.

### 2.9 Data analysis

Raw FASTQ files were processed using CellRanger (v6.0.2) to map reads against the human genome 38 as a reference. After alignment, in‐depth quality control, normalisation and analyses were conducted using Seurat (v4.0.5) in R (v4.4.2). Cell quality control thresholds were nFeature_RNA (100‒6000), percent.mt (0%, 15%), nCount_RNA (0, 60 000) and screened out outliers. We initially performed clustering using Seurat and identified NK cells as CD3D^−^ GNLY⁺. This subset was then extracted for further analysis. Within the NK cell population, we carried out principal component analysis, selecting the top 10 principal components for downstream analysis. We used the R package Harmony (v1.2.0)^25^ to remove batch effects caused by differences between patients. Clustering was then conducted on the Harmony‐corrected embedding with a resolution parameter of  .2. To identify cluster‐specific markers, differential expression was analysed for each cluster compared to all remaining cells and within each cluster. Differential expression within each cluster, determined by the Wilcoxon rank‐sum test. The gene set enrichment analysis (GESA) was applied to explore the differences in pathways.^26^

### 2.10 Statistical analysis

Baseline characteristics were assessed using descriptive statistical analysis, presented as mean and standard deviation or median (range) for continuous variables and compared using the *t*‐test and Wilcoxon Mann−Whitney test. When continuous variables simultaneously meet the conditions of homogeneity of variance and normal distribution, the *t*‐test was applied; otherwise, the Wilcoxon Mann‒Whitney test was used. All tests were two‐sided, with *p *< .05 considered statistically significant. Statistical analyses were performed using SPSS (v21.0, IBM Corporation). The image was drawn using GraphPad Prism (v8.0.2) and the immune‐related analysis was conducted using IORB.^27^

## 3 RESULTS

### 3.1 Seven subsets of NK cells identified by scRNA‐seq

**FIGURE 1:**
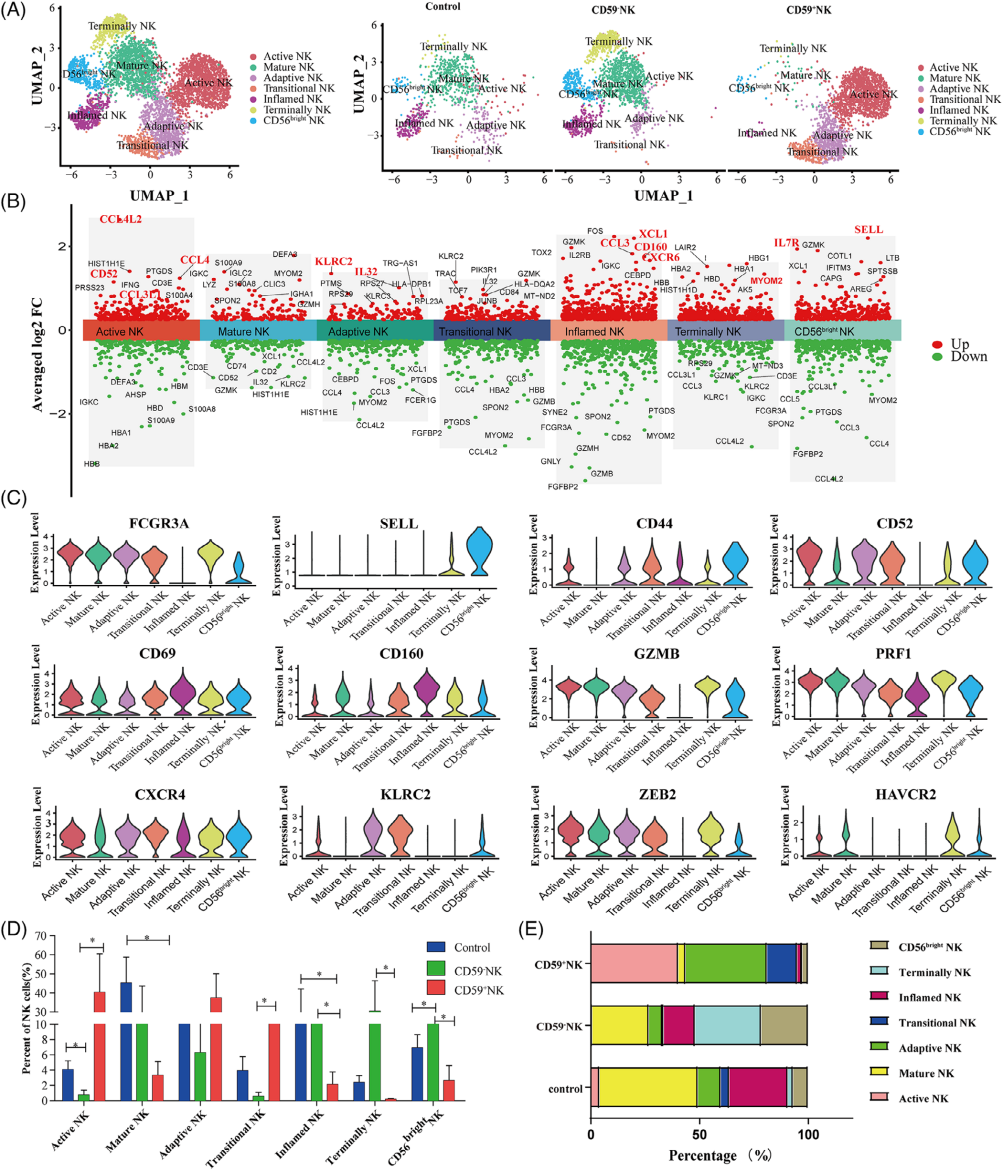
Clustering of human natural killer (NK) cells from bone marrow (BM). (A)Uniform manifold approximation and projection (UMAP) distribution of NK cells in BM (left), and the differences between patients with paroxysmal nocturnal haemoglobinuria (PNH) (N and P groups) and healthy controls (right). (B) sRNA‐SEQ volcano map is a comparison of multiple clusters (vs. other clusters), different clusters on the *x*‐axis and avg_log2FC on the *y*‐axis. (C) Violin plots of cell‐specific markers demonstrate the expression of NK‐lineage‐defining markers of each cluster. (D and E) Ratio of each observed NK cell type in the patients with PNH and healthy controls. ^*^ *p *< .05. Source data are provided as a Source Data file. See also Supporting Information tables.

In total, 130 120 cells were received and 7190 cells lost (5.5%), leaving 122 930 BMMNCs (38 820 CD59^−^ cells, 39 070 CD59^+^ cells and 45 040 control cells) for further analysis. We obtained 4640 NK cells from the BMMNCs (1676 CD59^−^ NK cells, 2186 CD59^+^ NK cells and 778 control NK cells), which were divided into seven blood‐derived cell clusters and visualised using a t‐distributed stochastic neighbour embedding (t‐SNE) diagram, where each dot represents a cell. Based on differentially expressed genes (DEGs) defining each cluster, we named them as ‘CD56^bright^ NK’, ‘transitional NK’, ‘active NK’, ‘adaptive NK’, ‘mature NK’, ‘terminal NK’ and ‘inflamed NK’ (Figure 1A). The t‐SNE diagram revealed clear differences among the three groups (Figure 1A). The sRNA‐SEQ volcano map and violin plots showed cell‐specific markers and demonstrated the expression of NK‐lineage‐defining markers in each cluster (Figure 1B,C). The ‘CD56^bright^ NK’ cluster had high *SELL*, *CD44* and *CD52* expressions, with lower *FCGR3A* expression. The ‘active NK’ cluster showed elevated levels of *CD52*, *CD69*, *CXCR4*, *PRF1*, *CXCR4* and *ZEB2* expressions. The ‘mature NK’ and ‘terminal NK’ clusters were characterised by high of *FCGR3A*, *GZMB* and *PRF1*. The ‘terminal NK’ clusters expressed *ZEB2* and *HAVCR2*. The ‘adaptive NK’ cluster had high *CD52*, *KLRC2*, *CXCR4* and *ZEB2* expressions. Unlike other NK cell clusters, the ‘inflamed NK’ cluster lacked *ZEB2*, *HAVCR2* and *CD52* expressions but expressed *CD69* and *CD160* (Figure 1C).

We analysed NK cell subgroups and found that active NK and adaptive NK cells increased in CD59^+^ NK cells, whereas CD56^bright^ NK and terminal NK increased in CD59^−^ NK cells, the mature NK cells decreased in both normal and abnormal clones compared to controls. Although adaptive NK cells increased in normal clones, the difference was not statistically significant compared to controls (Figure 1D,E and Table S2). To further understand the abnormal and normal NK clones, we analysed CD56^bright^ NK, active NK, terminal NK and mature NK cells.

### 3.2 Abnormal CD56^bright^ NK cells with reduced chemokine expression in classical PNH

**FIGURE 2:**
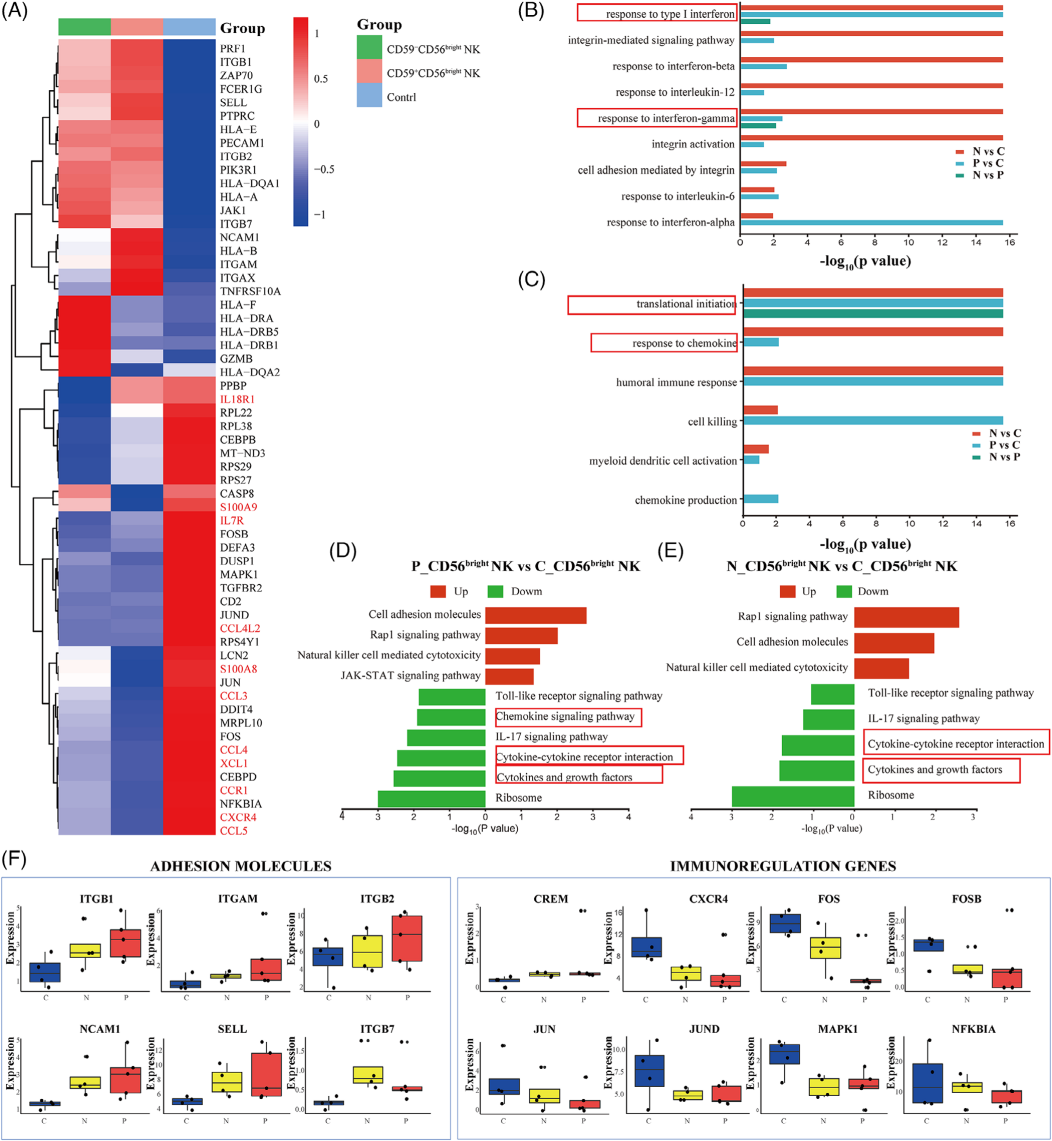
Single‐cell sequencing analysis of CD56^bright^ natural killer (NK) cells between paroxysmal nocturnal haemoglobinuria (PNH) and healthy controls. (A) Heatmap displaying the expression level of target genes of CD56^bright^ NK in CD59^−^CD56^bright^ NK, CD59^+^CD56^bright^ NK and control. (B and C) Gene ontology (GO) enrichment analysis of differentially expressed genes (DEGs) in up and downregulated pathways in pairwise comparison at three groups (B, upregulated pathways; C, downregulated pathways). (D and E) Kyoto Encyclopedia of Genes and Genomes (KEGG) enrichment analysis of DEGs in up and downregulated pathways in P and N groups compared with C groups (D, upregulated pathways; E, downregulated pathways). (F) Box plots showing expression of the different function genes of CD56^bright^ NK in three groups (N, CD59^−^CD56^bright^ NK; P, CD59^+^CD56^bright^ NK; C, control).

CD56^bright^ NK cells are a subtype of NK cells mainly involved in immune regulation, characterised by high cytokine production capacity and low cytotoxic activity. Our analysis revealed a significant increase in the number of CD59^−^CD56^bright^ NK cells across the three groups. DEGs showed that CD59^−/+^CD56^bright^ NK cells have downregulated inflammatory cytokine‐related genes (*XCL1*, *CCL3*, *CCL4*, *CCL5*, *CCL4L2* and *CXCR4*) compared to controls (Figure 2A). Notably, recent studies suggest that NK cell‐derived XCL1 plays a crucial role in recruiting dendritic cells to the tumour microenvironment, which is critical for antitumour immunity.^16^ Gene ontology (GO) enrichment analysis showed that CD59^+/−^CD56^bright^ NK cells have upregulated type I IFN (Figure 2B), and downregulated translational initiation compared to controls (Figure 2C). Functional analyses suggest that CD56^bright^ NK cells are defective in patients with PNH. Kyoto Encyclopedia of Genes and Genomes (KEGG) enrichment analysis showed that CD59^‒/+^CD56^bright^ NK cells have upregulated NK cell‐mediated cytotoxicity and the Rap1 and JAK‒STAT signalling pathways—both of which are critical for cell proliferation, differentiation and apoptosis compared to controls. Conversely, these cells have downregulated cytokine‐ and chemokine‐related signalling pathways compared to controls (Figure 2D,E). Additionally, immunoregulatory genes expression was downregulated in CD59^+/−^CD56^bright^ NK cells (Figure 2F). These results suggest that CD56^bright^ NK cells in patients with PNH may exhibit enhanced responsiveness to cytokine stimulation. However, the downregulation of cytokine‐ and chemokine‐related genes may impair their regulatory function.

### 3.3 Increased CD59^+^ active NK in patients with PNH with enhanced cytotoxic function and cytokine responses

**FIGURE 3:**
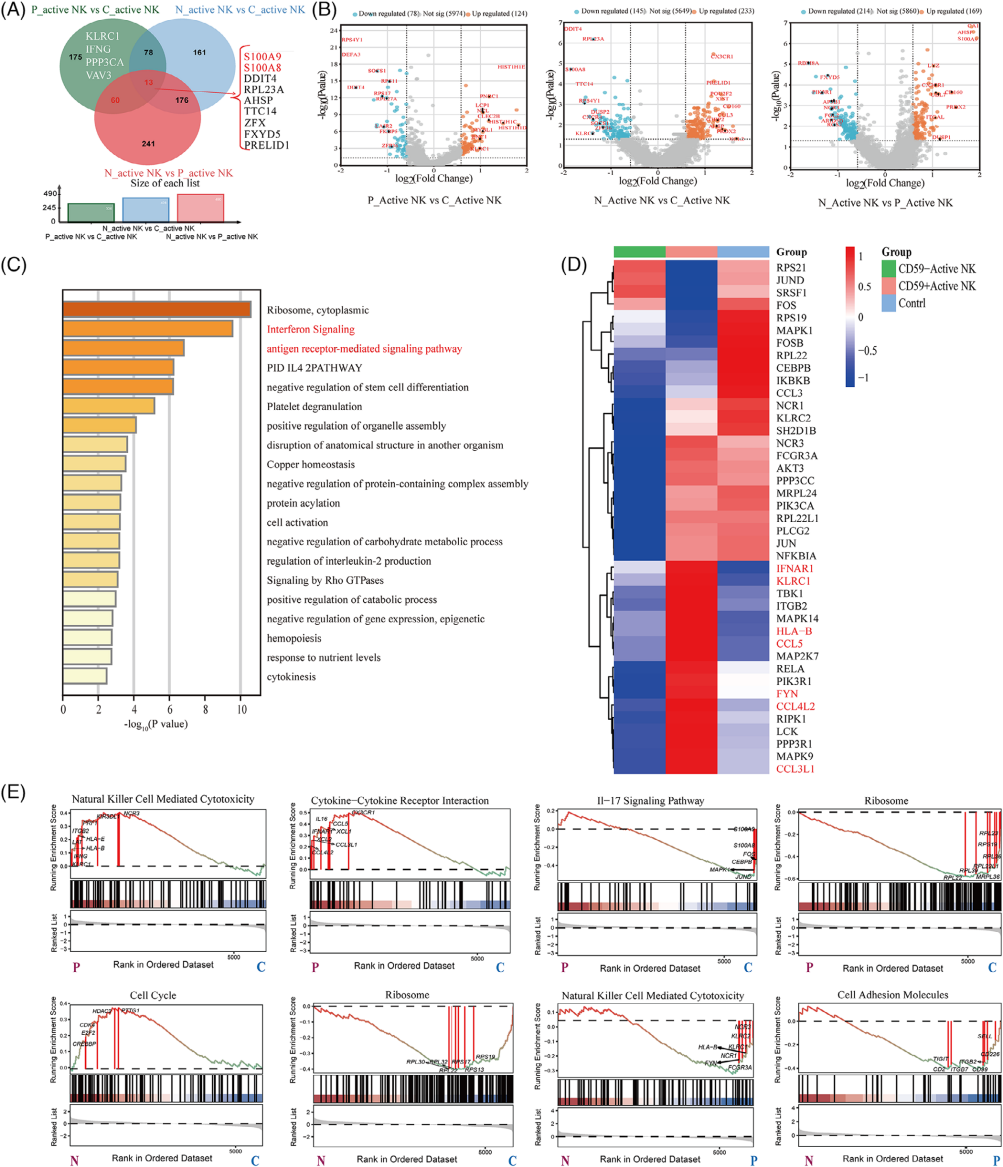
Single‐cell sequencing analysis of active natural killer (NK) cells between paroxysmal nocturnal haemoglobinuria (PNH) and healthy controls. (A) Venn diagram shows the differentially expressed genes (DEGs) at three groups (Wilcoxon rank‐sum test, absolute log2FC ≥.5, *p*‐value <.05). (B) Volcano plots shows the DEGs (left, P_active compared to C_active; middle, N_active compared to C_active; right, N_active compared to P_active). (C) Differential gene enrichment analysis between CD59^+/−^ active NK and control. (D) Heatmap displaying the expression level of target genes of active NK in three group. (E) Selected gene sets enriched in the ‘active NK’ cluster of three pairwise comparison groups (P, CD59^+^ active NK cells; N, CD59^−^ active NK cells; C, controls).

Active NK cells represent an activated state of NK cells, likely reflecting their homeostatic activation in response to certain stimuli. These cells may exert considerable effects on NK cell survival, proliferation and differentiation. Studies also suggest that active NK cells may indicate NK cell exhaustion.^28^ The proportion of CD59^+^ active NK cells was significantly higher than that of CD59^─^ active NK and control cells (Figure 1D). A Venn diagram showing DEGs identified through pairwise comparisons among CD59^+^ active NK cells, CD59^−^ active NK cells and the control cells (Figure 3A). Thirteen genes overlapped among the three groups, KLRC3, IFNG, PPP3CA and VAV3 were significantly upregulated CD59^+^ active NK cells. A total of 202 DEGs were identified through pairwise comparisons among the three groups (Wilcoxon rank‐sum test, absolute log2FC ≥.5, *p‐*value <.05), including 78 upregulated genes and 124 downregulated genes in CD59^+^ active NK cells of patients with PNH (Figure 3B). DEG enrichment analysis revealed that CD59^+/─^ active NK cells were mainly enriched in IFN‐mediated and antigen receptor‐mediated signalling pathways compared to controls (Figure 3C). A heatmap of DEGs showed that CD59^+^ active NK cells expressed high levels of NK cytotoxicity‐related genes (*KLRC1*, *IFNAR1*, *HLA‐B*, *PRF1*, *LAT* and *FYN*) and chemokine genes (*CCL5*, *CCL4L2* and *CCL3L1*), whereas most genes in CD59^−^ active NK cells were downregulated (Figure 3D). KEGG enrichment analysis showed that CD59^+^ active NK cells have significantly upregulated NK cell‐mediated cytotoxicity and cytokine‐cytokine receptor interactions, while downregulated the IL‐17 signalling pathway and ribosomal pathways compared to controls. Compared to CD59^+^ active NK cells, CD59^−^ active NK cells have downregulated NK cell‐mediated cytotoxicity (Figure 3E). These results suggest that CD59^+^ active NK cells are more reactive in patients with PNH, exhibiting enhanced cytotoxicity and cytokine production compared to CD59^−^ active NK cells and controls.

### 3.4 Reduced mature NK cells in both normal and abnormal clones in patients with PNH

**FIGURE 4:**
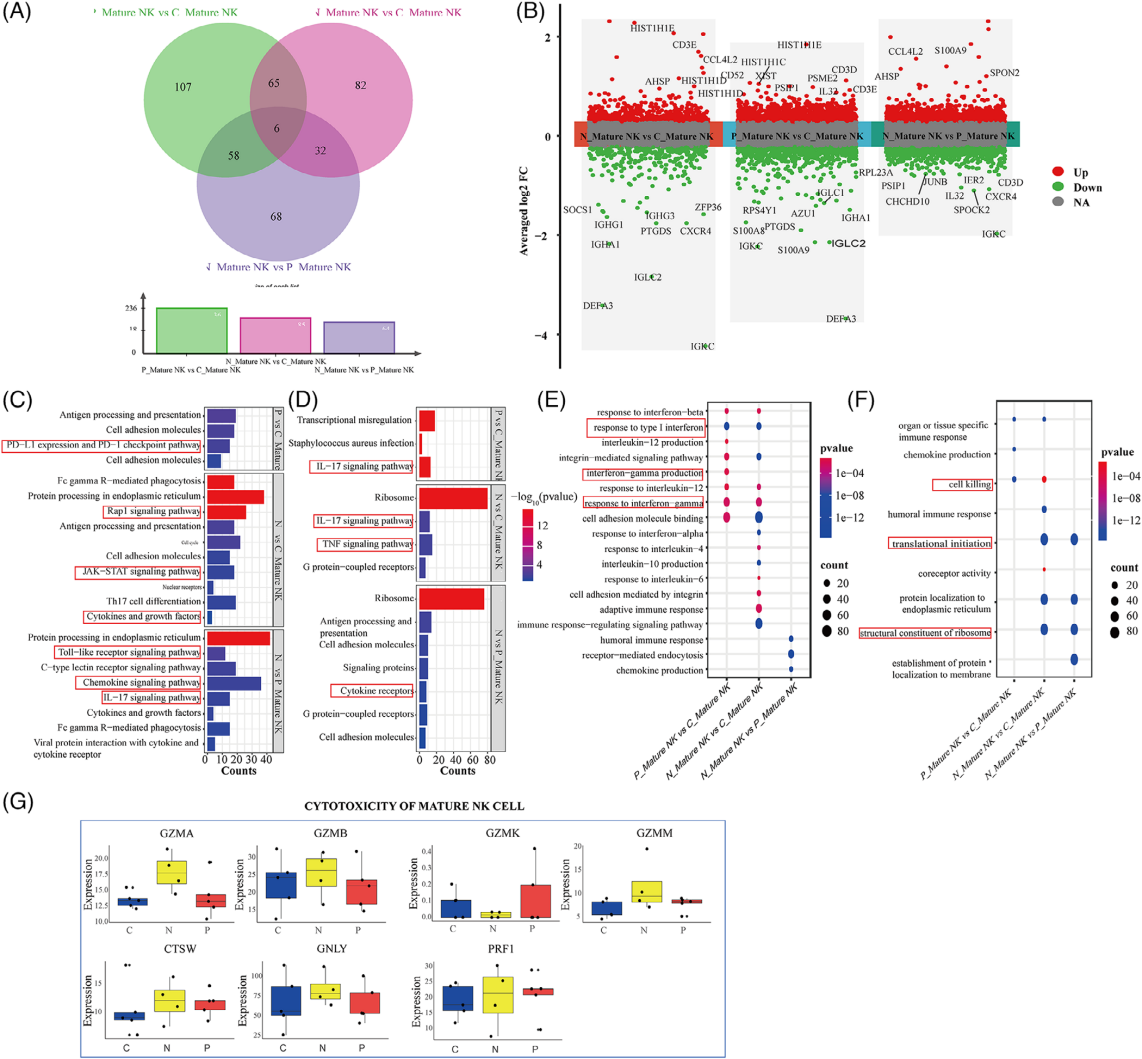
Single‐cell sequencing analysis of mature natural killer (NK) cells between paroxysmal nocturnal haemoglobinuria (PNH) and healthy controls. (A) Venn diagram shows the differentially expressed genes (DEGs) at three groups (Wilcoxon rank‐sum test, absolute log2FC ≥.5, *p*‐value <.05). (B) sRNA‐SEQ volcano map is a pairwise comparison at three groups in mature NK cells, different groups on the *x*‐axis and averaged_log2FC on the *y*‐axis. (C and D) Kyoto Encyclopedia of Genes and Genomes (KEGG) enrichment analysis of DEGs in up and downregulated pathways in pairwise comparison at three groups (C, upregulated pathways; D, downregulated pathways). (E and F) Gene ontology (GO) enrichment analysis of DEGs in up and downregulated pathways in pairwise comparison at three groups (E, upregulated pathways; F, downregulated pathways). (G) Box plots showing expression of the cytotoxicity genes of mature NK in three groups (N, CD59^−^ mature NK; P, CD59^+^ mature NK; C, control).

Mature NK cells exhibit strong cytotoxicity, rapidly initiating killing mechanism without prior sensitisation. They release perforin, granzyme and other substances to directly destroy target cells and secrete cytokines, such as IFN‐γ. The number of mature NK cells in both normal and abnormal clones was significantly decreased in patients with PNH (Figure 1D,E). A Venn diagram shows the DEGs detected by pairwise comparison of CD59^+^ mature NK cells, CD59^−^ mature NK cells and controls (Figure 4A). DEG analysis showed that CD59^−^ mature NK cells upregulated *CCL4L2* and downregulated immunoglobulin genes, chemokine gene *CXCR4*, and immune regulation‐related genes *DEFA3* compared to controls. CD59^+^ mature NK cells upregulated *CD52*, *IL32* and *PSME2* while downregulating *S100A8*, *S100A9* and *DEFA3* compared to controls (Figure 4B). *DEFA3* primarily contributes to immune regulation. *PSME2* encodes a proteasome activator subunit 2, encoding a protein responsible for activating the proteasome, which is essential for maintaining cell function. In the immune system, *PSME2* participates in antigen presentation and T‐cell activation, enhancing the immune response. KEGG enrichment analysis indicated that CD59^−^ mature NK cells have upregulated the Rap1 and JAK‒STAT signalling pathways, which involved in cell proliferation and differentiation, and cytokine‐related signalling pathways, while have downregulated the IL‐17 and tumor necrosis factor (TNF) signalling pathways (Figure 4C,D). GO enrichment analysis suggested that CD59^‒/+^ mature NK cells have upregulated the IFN signalling pathway while downregulated transcriptional activation and ribosome‐related processes compared to controls (Figure 4E,F). Genes associated with cytotoxicity showed no significant difference among the three groups (Figure 4G). These results suggest that CD59^−/+^ mature NK cells have an enhanced response to IFN signalling but downregulating some immunomodulatory genes, especially those in the IL‐17 and TNF signalling pathways.

### 3.5 CD59^−^ terminal NK cells are increased in patients with PNH

**FIGURE 5:**
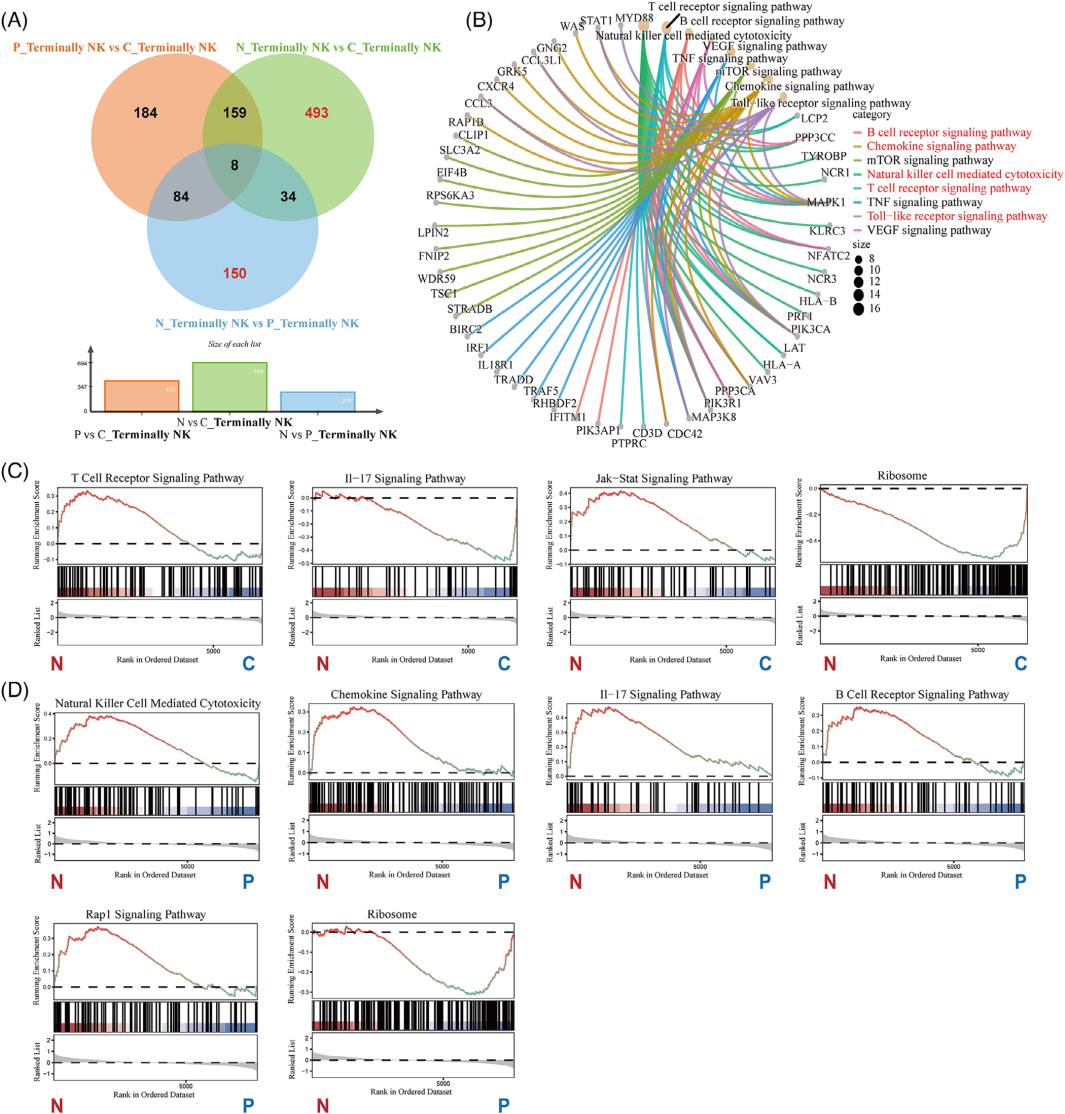
Single‐cell sequencing analysis of terminally natural killer (NK) cells between paroxysmal nocturnal haemoglobinuria (PNH) and healthy controls. (A) Venn diagram shows the differentially expressed genes (DEGs) at three groups (Wilcoxon rank‐sum test, absolute log2FC ≥.5, *p*‐value <.05). (B) Cnet diagram shows DEGs enrichment pathways between CD59^−^ terminal NK and CD59^+^ terminal NK and control. (C) GESA analysis between CD59^−^ terminal NK and control. (D) GESA analysis between CD59^−^ terminal NK and CD59^−^ terminal NK (N, CD59^−^ terminal NK; P, CD59^+^ terminal NK; C, control).

Terminal NK cells express similar functional molecules as mature NK cells. Previous studies reported that terminal NK cells downregulate the inflammatory response, mTOR/STAT5 signalling and metabolic activity, suggesting a quiescent state.^29^ In this study, the proportion of CD59^‒^ terminal NK cells was significantly increased (Figure 1D,E). The Venn diagram revealed 493 DGEs between CD59^‒^ terminal NK cells and controls and 150 DGEs between CD59^‒^ and CD59^+^ terminal NK cells (Figure 5A). KEGG enrichment analysis showed that these DEGs were mainly associated with NK cell‐mediated cytotoxicity, the B cell receptor ‐ T cell receptor (BCR‒TCR), chemokine, and Toll‐like receptor signalling pathway (Figure 5B). GESA analysis showed that CD59^‒^ terminal NK cells have upregulated the T‐cell receptor, IL‐17 and JAK‒STAT signalling pathway while downregulated ribosomes compared with controls (Figure 5C). Compared with CD59^+^ terminal NK cells, CD59^‒^ terminal NK cells have upregulated NK cell‐mediated cytotoxicity, chemokine, IL‐17, B‐cell receptor and Rap1 signalling pathways while downregulated ribosomes (Figure 5D). These results indicate that CD59^−^ terminal NK cells are increased in both number and function in patients with classical PNH.

### 3.6 NK cell subgroup verified by flow cytometry aligns with those of scRNA‐seq

**FIGURE 6:**
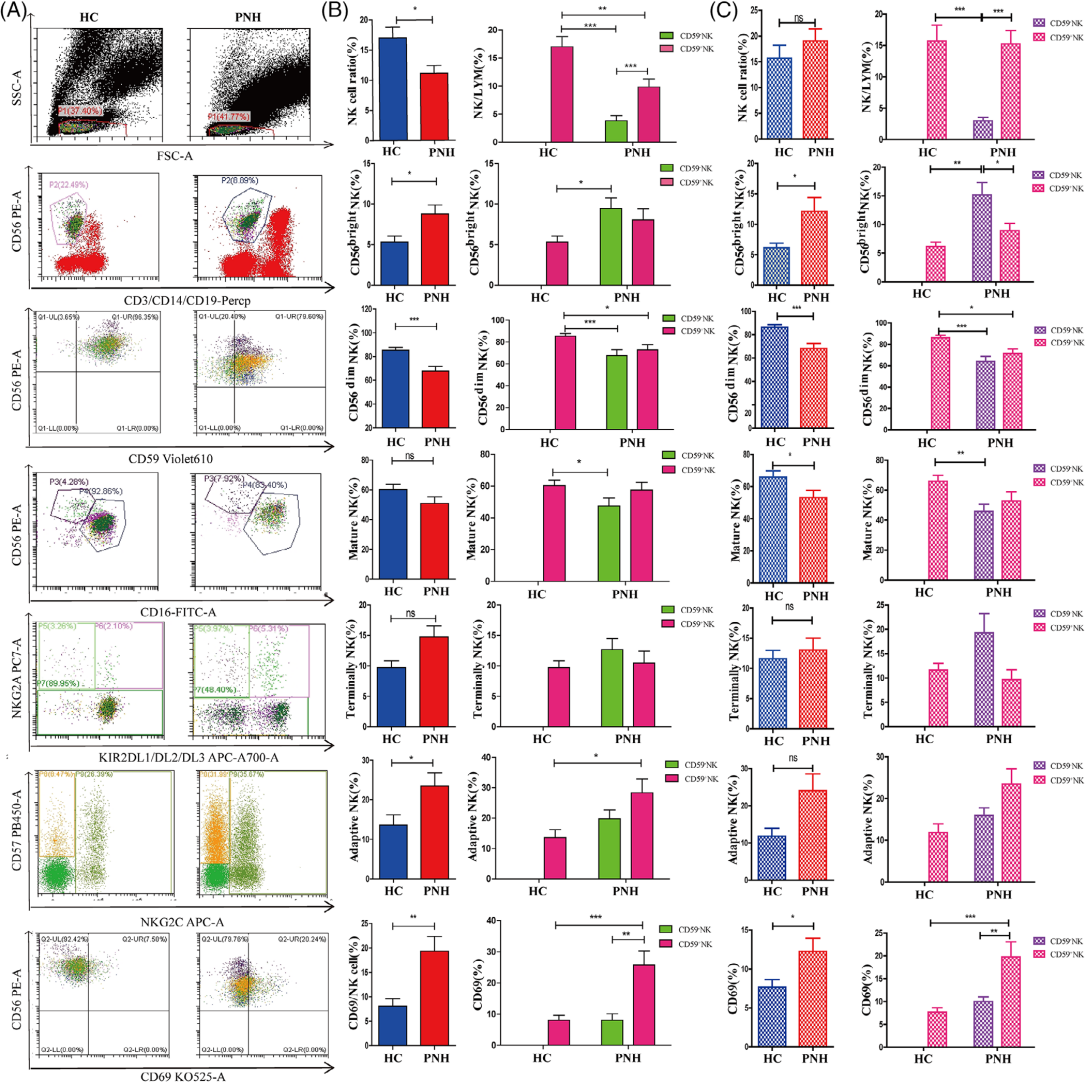
Natural killer (NK) cells were detected by flow cytometry in peripheral blood (PB) and bone marrow (BM) of patients with paroxysmal nocturnal haemoglobinuria (PNH). (A) FCM plots are representative examples and show the NK cells and NK cell subsets were detected by flow cytometry in healthy control (HC) and PNH. (B) Proportion of NK cells and the proportion of each subgroup were statistically analysed in PB. (C) Proportion of NK cells and the proportion of each subgroup were statistically analysed in BM. ^*^ *p *< .05; ^**^ *p *< .01; ^***^ *p *< .001; n.s. stands for ‘not significant’. Data information is provided in the Supporting Information tables.

To further validate the scRNA‐seq results, we used flow cytometry (FCM) to assess the proportion and subgroup distribution of NK cells in the BM and peripheral blood of patients with classical PNH and healthy individuals (Figure 6A). In peripheral blood, the proportion of NK cells in patients with PNH (11.13 ± 6.55%) was significantly lower than that in the HCs (17.00 ± 9.53%) (*p *= .01). In patients with PNH, CD59^+^ NK cells constitute the predominant population in PNH (*p *< .001). The proportion of CD56^bright^ NK cells was significantly higher in patients with classical PNH than in HCs ([8.80 ± 5.55%] vs. [5.32 ± 3.87%], *p *= .01), with a notable trend towards an increase in CD59^‒^CD56^bright^ NK cells. CD56^dim^ NK cells were significantly reduced compared to HCs (71.51 [51.40, 86.13]% vs. 88.53 [81.97, 94.68]%, *p *< .001). There were no significant differences in the proportions of mature and terminal NK cells between PNH and HC. The proportion of adaptive NK cells in patients with PNH was significantly higher than in HCs (*p *= .03), with a similarly significant increase in CD59^+^ adaptive NK cells (*p *= .03). To assess NK cell activation in patients with PNH, we measured CD69 expression on the NK cell surface, which was significantly increased in patients with PNH than that in HCs (*p *= .002), with higher expression observed in normal clones (Figure 6B and Tables S3 and S4).

There was no significant difference in the proportion of NK cells between PNH patients and HCs in the BM (*p *= .18), and CD59^+^ NK cells constitute the predominant population in PNH (*p *< .001). The proportion of CD56^bright^ NK cells in the BM of patients with PNH was significantly higher than in HCs (*p *= .02), and CD59^‒^CD56^bright^ NK cells accounted for the majority in PNH (*p *= .02). Conversely, the proportions of CD56^dim^ NK cells (*p *< .001) and mature NK cells (*p *= .04) in patients with PNH were significantly lower than in HC, with fewer abnormal clones in PNH. There were no significant differences in the proportion of terminal NK cells (*p *= .54) or adaptive NK cells (*p *= .10) between groups; however, an increase trend towards was observed in CD59^‒^ terminal NK and CD59^+^ adaptive NK cells. CD69 expression in NK cells was significantly higher than that in HCs (*p *= .02). These flow cytometry results confirmed the scRNA‐seq findings that CD56^bright^ NK cells are expanded, CD56^dim^ and mature NK cells are reduced, and CD69 expression is elevated in patients with PNH (Figure 6C and Tables S5 and S6).

### 3.7 NK cells may be involved in T‐cell regulation and PNH clonal immune escape mechanism

We analysed peripheral blood T lymphocyte subsets in 24 patients with classical PNH and 12 HCs (Figure S1A). The results showed that the proportion of CD8^+^ T cells (41.83 ± 8.31%) was significantly higher than HCs (34.45 ± 12.03%) (*p *= .04). Conversely, the proportion of CD4^+^ T cells (34.79 ± 8.10%) was lower than HCs (42.40 ± 13.19%), although the difference was not statistically significant (*p *= .09). However, the CD4/CD8 ratio (.86 ± .32) was significantly lower than HC (1.44 ± .81) (*p *= .03) (Figure S1B).

The proportion of NK cells was negatively correlated with CD8^+^ T‐cell proportion (*R* ^2^ = ‒.67, *p *< .001), lactate dehydrogenase (LDH) level (*R* ^2^ = ‒.51, *p *= .007), reticulocyte % (*R* ^2^ = ‒.63, *p *= .001), reticulocyte absolute value (*R* ^2^ = ‒.46, *p *= .017) and free haemoglobin (*R* ^2^ = ‒.64, *p <* .001). Conversely, NK cell proportion was positively correlated with the CD4/CD8 ratio (*R* ^2^ = .61, *p *= .001). No significant correlation was observed with CD4^+^ T‐cell proportion, red blood cells, haemoglobin, white blood cell count, platelet count, PNH clone size or bilirubin levels (Figure S1C).

### 3.8 Functional impairment of CD59^−^ NK cells in PNH, with minimal change in CD59^+^ NK cells

**FIGURE 7:**
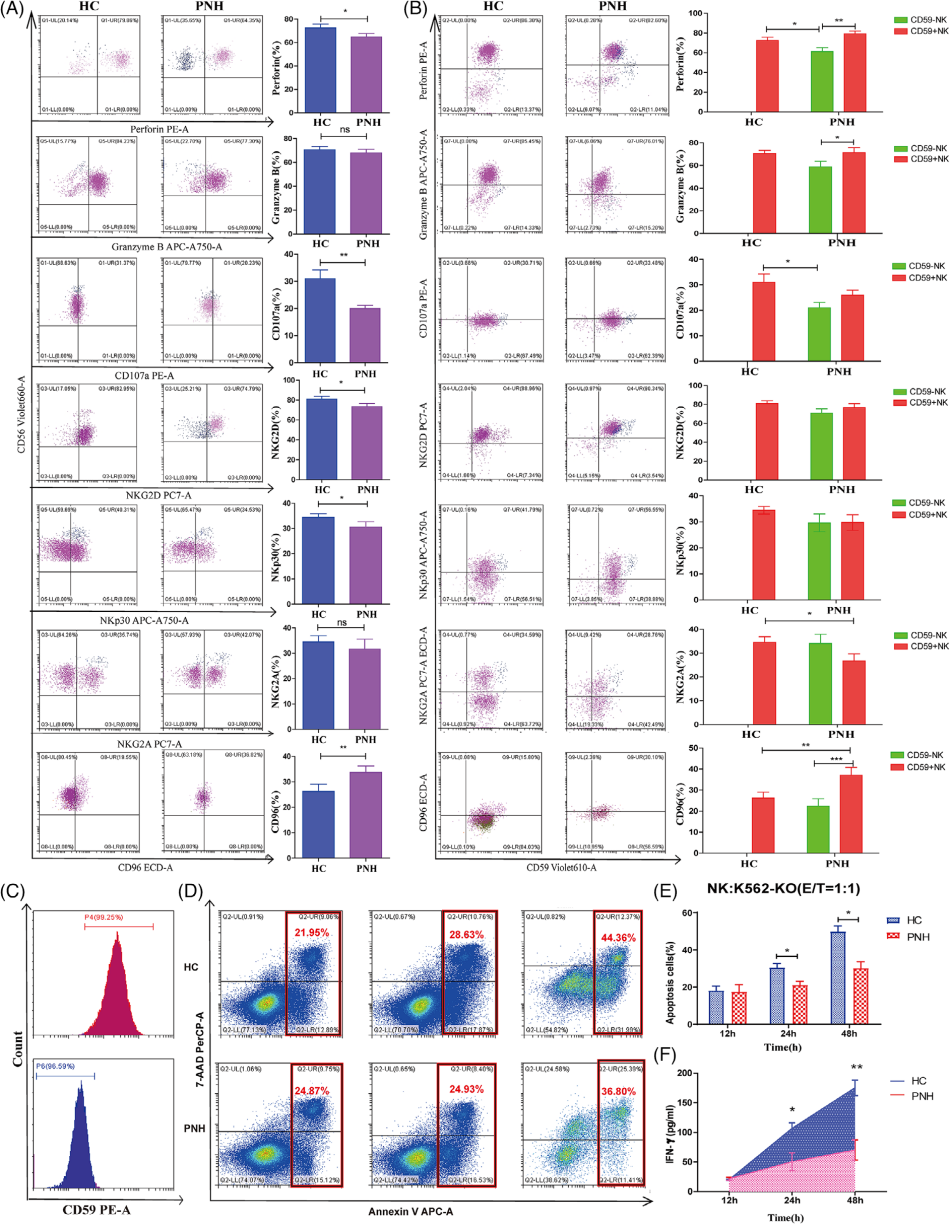
Function of natural killer (NK) cells were detected in paroxysmal nocturnal haemoglobinuria (PNH) patients and control. (A) FCM plots are representative examples and function molecules of NK cells were detected by flow cytometry and statistically analysed. (B) FCM plots are representative examples and function molecules of NK cells were compared in CD59^−^ NK, CD59^+^ NK and healthy control (HC). (C) Glycosyl‐phosphatidylinositol (GPI) anchored protein (GPI‐AP) (CD59) was detected in K562 cell lines (upper) and knockout K562 cell line (K562‐KO) cell lines (under). (D) Apoptosis rate of K562‐KO cells at 12, 24 and 48 h was detected by flow cytometry after co‐culture with NK cells. (E) Apoptosis rate of K562‐KO was statistically analysed. (F) Level of interferon‐γ (IFN‐γ) secretion of co‐culture supernatant was detected by enzyme‐linked immunosorbent assay (ELISA). ^*^ *p *< .05; ^***^ *p *< .01; ^***^ *p *< .001; n.s. stands for ‘not significant’. Data information is provided in the Supporting Information tables.

The expression of surface‐activated receptors (NKG2D and NKp30), inhibitory receptors (NKG2A and CD96), degranulation markers (CD107a) and cytotoxic granules (Perforin, Granzyme B) was detected using flow cytometry (Figure 7A). NK cells were divided into normal and abnormal clones using CD59 expression, and the expression of these molecules were analysed (Figure 7B). The results showed expression levels of perforin (*p *= .013) and CD107a (*p *= .005) were significantly reduced in patients with PNH. The expression level of NKG2D was significantly decreased (80.25 [68.06, 86.86]% vs. 87.30 [74.14, 92.36]%, *p *= .031), and expression level of CD96 was significantly increased (33.58 [26.55, 41.40]% vs. 24.43 [16.49, 31.17]%, *p *= .001) (Table S7).

The expression level of perforin in CD59^−^ NK cells was significantly lower than HCs (*p *= .009) and CD59^+^ NK cells (*p *< .001). Similarly, the expression of granzyme B in CD59^−^ NK cells was significantly lower than CD59^+^ NK cells (*p *= .048). The expression of CD107a in CD59^−^ NK cells was significantly lower than HCs (*p *= .019). The expression of NKG2A in CD59^+^ NK cells was lower than HCs (*p *= .048). The expression of CD96 in CD59^−^ NK cells was significantly lower than CD59^+^ NK cells (*p *< .01), and the expression of CD96 in CD59^+^ NK cells was significantly higher than HCs (*p *= .002) (Table S8).

A co‐culture experiment involving NK and K562‐KO cells further confirmed the cytotoxic function of NK cells in patients with classical PNH (Figure 7D). The co‐culture results showed that the apoptosis rate of K562‐KO in patients with PNH (17.50 ± 6.60%) and HCs (18.18 ± 4.18%) was not difference significantly (*p *= .89) after 12 h of co‐culture. However, at 24 h, the apoptosis rate in patients with PNH (21.16 ± 3.47%) was significantly lower than HCs (30.49 ± 3.78%) (*p *= .035). By 48 h, this difference became more pronounced (*p *= .012) (Figure 7E). Similarly, analysis of co‐culture supernatants revealed that IFN‐γ levels followed the same trend (Figure 7F). These results suggest that NK cell function is impaired in patients with PNH.

### 3.9 Changes in NK cell number and function verified in a PNH animal model

**FIGURE 8:**
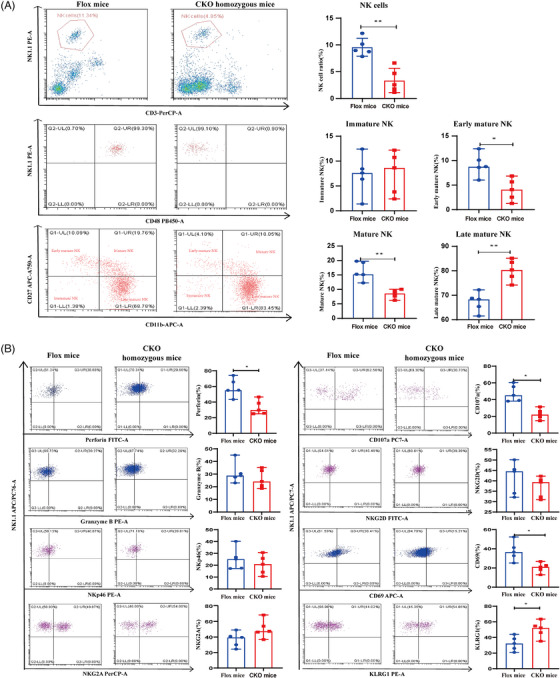
Proportion and function of natural killer (NK) cells were detected in Flox mice and CKO homozygous mice. (A) FCM plots were representative examples and proportion of NK cells and NK cell subsets were detected by flow cytometry in peripheral blood. (B) FCM plots were representative examples and function molecules of NK cells were detected by flow cytometry. ^*^ *p *< .05; ^**^ *p *< .01; ^***^ *p *< .001; n.s. stands for ‘not significant’. Data information is provided in the Supporting Information tables.

We have constructed a PIGA conditional knockout mouse model using the ES targeting technique and Vav‐iCre mice.^30^ The expression of GPI and GPI‐AP (CD48) on NK cells was almost completely absent in CKO homozygous mice (Figure 8A). The proportion of NK cells in peripheral blood of CKO homozygous mice (3.56 [1.46, 5.66]%) was significantly lower than that in flox mice (8.97 [8.30, 11.16]%, *p *= .01). Compared with flox mice, CKO homozygous mice showed a significant decrease in the proportion of early mature NK cells (*p *= .02) and mature NK cells (*p *= .01), and an increase in the proportion of late mature NK cells (*p *= .01) (Figure 8A and Table S9). The proportion of NK cells was negatively correlated with the LDH level (*R* ^2^ = ‒.94, *p *= .02) in CKO homozygous mice. However, there was no correlation with white blood cell count, platelet count, red blood cell count, haemoglobin level, hematocrit (HCT), neutrophil ratio, lymphocyte ratio and bilirubin level (*p *> .05) (Figure S2).

We compared functional molecules between the two groups and found significantly decreased perforin (*p *= .02) and CD107a (*p *= .01) expression in the NK cells of CKO homozygous mice, with no significant difference in granzyme B expression (*p *= .47). CD69 expression was significantly decreased (*p *= .02), whereas the expression of KLRG1 was significantly increased (*p *= .03). No significant differences were observed in NKG2D, NKp46 and NKG2A expression (*p *> .05) (Figure 8B and Table S10).

## 4 DISCUSSION

PNH is an ultra‐rare acquired clonal disorder which is characterised by intravascular haemolysis, thrombosis and varying degrees of BM failure, with a 35% mortality rate in patients receiving only supportive treatment.^31^ While complement inhibitors significantly improve haemoglobin levels, control haemolysis, reduce complications and enhance the quality of life, they do not cure PNH or eradicate PNH clones. Despite advancements in PNH diagnosis and treatment, it remains unclear how PNH clones, arising from mutated HSCs, expand in the BM. Studies have suggested that immune escape,^32^ anti‐apoptotic mechanism^33^ and secondary gene mutation^34^, ^35^ contribute to PNH clone proliferation. However, the immune microenvironment of BM cells in patients with PNH remains incompletely understood.

Previous investigations have predominantly focused on the differences in the impacts of NK cells on normal and abnormal clonal cells, particularly the mechanisms by which PNH clonal cells evade immune responses. Nevertheless, there has been a paucity of research regarding potential differences among the NK cells of PNH patients and the functions of normal clonal NK cells and abnormal clonal NK cells. We conducted scRNA‐seq of BMMNCs from patients with PNH, distinguishing the roles of normal and abnormal NK cell clones in PNH for the first time. Previous research has indicated that activated T and NK cells collectively contribute to immune escape in PNH.^23^ Therefore, NK cells from patients with PNH were further analysed in this study. The researches have revealed that patients with PNH have defects in NK cells, manifesting the absolute number and proportion of NK cells in the blood of patients with PNH are lower than those in healthy individuals.^19^, ^20^, ^36^ However, the changes in function of NK cells were controversy. El‐Sherbiny et al.^37^ believed that GPI anchor chain protein plays a significant role in maintaining the stability of NK cell subpopulations, and there is a defect in the chemotactic function of GPI‐NK cells. The research have also shown that adaptive NK cells exist independently in patients with PNH and may play an important role in the pathogenesis of PNH clonal immunity.^38^ Our research is different from previous studies. For the first time, we utilised single‐cell sequencing technology to analyse the differences in NK cells and their subgroups between patients with classic PNH and healthy individuals. Second, we also conducted in vitro experiments and animal models to verify the function of NK cells in PNH patients, and explored the functional changes of normal and abnormal clonal NK cells.

The scRNA‐seq results revealed impaired function of CD59^−^ NK cells in patients with classical PNH, while CD59^+^ NK cells exhibited increased reactivity. The proportion and functional activity of NK cells in the peripheral blood of patients with PNH was decreased, which was closely related to clinical indicators (LDH, reticulocyte and free haemoglobin) and T lymphocytes. In vitro co‐culture experiments confirmed decreased NK cell function in classical PNH, suggesting weakened T‐cell regulation, leading to hyper‐function of T lymphocytes and potentially contributing to immune escape.

scRNA‐seq results indicated a significant increase in the proportion of CD59^+^ active NK cells, as well as CD59^−^CD56^bright^ NK and CD59^−^ terminal NK cells compared to controls. Conversely, the proportions of CD59^−^ mature NK and CD59^+^ mature NK were significantly decreased in patients with classical PNH. These results were also confirmed using flow cytometry. The differences in the distribution of CD59^+^ NK and CD59^−^ NK cell subsets suggest that GPI‐linked proteins play a role in maintaining NK cell subset homeostasis in classical PNH. The expression of chemokine genes in CD56^bright^ NK is decreased in patients with PNH, leading to impaired immune regulation. A study has also reported that the proportion of GPI^−^CD56^bright^ NK cells in patients with PNH is significantly higher than GPI^+^CD56^bright^ NK cells. Additionally, chemokine receptor expression differed between GPI^+^ and GPI^−^ NK cells, resulting in impaired chemotactic responses.^39^

Both CD59^+^ NK and CD59^−^ NK cells exhibit a significant reduction in ribosomal component gene expression, as well as in the IL‐17 signalling pathway and transcription factor levels. This suggests that NK cells in patients with PNH may have qualitative abnormalities associated with clonal immune escape. IL‐17 is a proinflammatory cytokine produced by T helper cell 17 that induces inflammatory factors and chemokines, direct or indirectly mediating the inflammatory response.^40^ IL‐17 also contributes to the immunopathology of autoimmune and chronic inflammatory diseases. Studies have shown that IL‐17 and its receptors are associated with autoimmune diseases, such as plaque psoriasis,^41^ autoimmune encephalomyelitis^42^ and multiple sclerosis.^43^ The IL‐17 signalling pathway plays an important role in immune regulation, particularly in inflammatory and immune responses. In our previous study, we found that IL‐17A levels were significantly decreased in patients with PNH.^44^ Therefore, we speculated that the IL‐17 signalling pathway may play an important role in immune regulation involving NK cells. Ribosomes are the main organelles involved in cellular protein synthesis. In addition, some ribosomal proteins (RP) have extra‐ribosomal functions in addition to protein biosynthesis, such as differentiation, proliferation and cell function.^45^ For example, *RPL5*, *RPL11*, *RPL23*, *RPS7* and *RPS26* induce apoptosis via the RP‐MDM2‐P53 pathway.^45^ Loss of *RPS19* impairs erythroid differentiation in Diamond‐Blackfan anaemia.^46^ Deficiency of *RPS14* haplotype may impaired cell maturation in 5q syndrome.^47^ Loss of *RPL22* initiates ribosomal stress, induces αβ‐T‐cell apoptosis in positive selection by activating P53, and impairs their maturation.^48^ *RPL22* is essential for normal lymphocyte development, and its loss may lead to the malignant transformation of T cells.^49^ Studies have also shown that NK cells with low ribosome expression decreased CD107a expression and impaired function.^50^ In this study, we found that many RP genes were downregulated or absent in patients with PNH. We speculate that restricted protein synthesis may impair NK cell function in PNH.

The main limitation of this article is the small number of NK cells obtained for scRNA‐seq, which may lead to potential biases during analysis. Furthermore, limited functional molecular assays may cause a deviation in the research results. In the future, we should explore the reasons for the changes in NK cells in PNH and whether improving NK cytotoxicity can reduce PNH clone expansion.

## 5 CONCLUSIONS

In patients with classical PNH, both the quantity and function of NK cells are reduced, with significant differences in the distribution of CD59^+^ and CD59^−^ NK cell subsets. This study revealed that CD59^+^ NK cells are mainly activated and primarily involved in immune escape, whereas CD59^−^ NK cells have functional defects in PNH, providing novel insights into NK cell dysfunction in PNH and highlighting their potential role in immune escape.

## AUTHOR CONTRIBUTIONS

*Conceptualisation*: Rong Fu and Hui Liu. *Methodology*: Chaomeng Wang, Yan Yang and Zhaoyun Liu. *Visualisation*: Wei Wang. *Funding acquisition*: Rong Fu. *Project administration and supervision*: Mengting Che, Honglei Wang, Lijuan Li, Yingying Chen and Liyan Li. *Writing—original draft*: Chaomeng Wang and Yan Yang. *Writing—review and editing*: Hui Liu and Rong Fu.

## CONFLICT OF INTEREST STATEMENT

The authors declare they have no conflicts of interest.

## ETHICS STATEMENT

This study was approved by the Ethics Committee of the Tianjin Medical University General Hospital (ethical no. IRB2025‐YX‐154‐01). All participants provided informed consent. Animal experiments were approved by the Animal Ethics Committee of the Tianjin Medical University General Hospital (ethical no. IRB2025‐DW‐35).

## Supporting information



Supporting Information

Supporting Information

Supporting Information

Supporting Information

Supporting Information

## Data Availability

The single‐cell RNA sequencing data have been deposited into Genome Sequence Archive(GSA) for Human with accession number HRA014654 (https://ngdc.cncb.ac.cn/gsa‐human/browse/HRA014654). All data are available in the main text or the Supporting Information.
