# Supplementary figures and images for: Role of NK cells in immune escape in patients with classical paroxysmal nocturnal haemoglobinuria
Source: Clin Transl Med. 2025 Dec 17;15(12):e70542. doi: 10.1002/ctm2.70542 (PMC12710432; doi:10.1002/ctm2.70542)

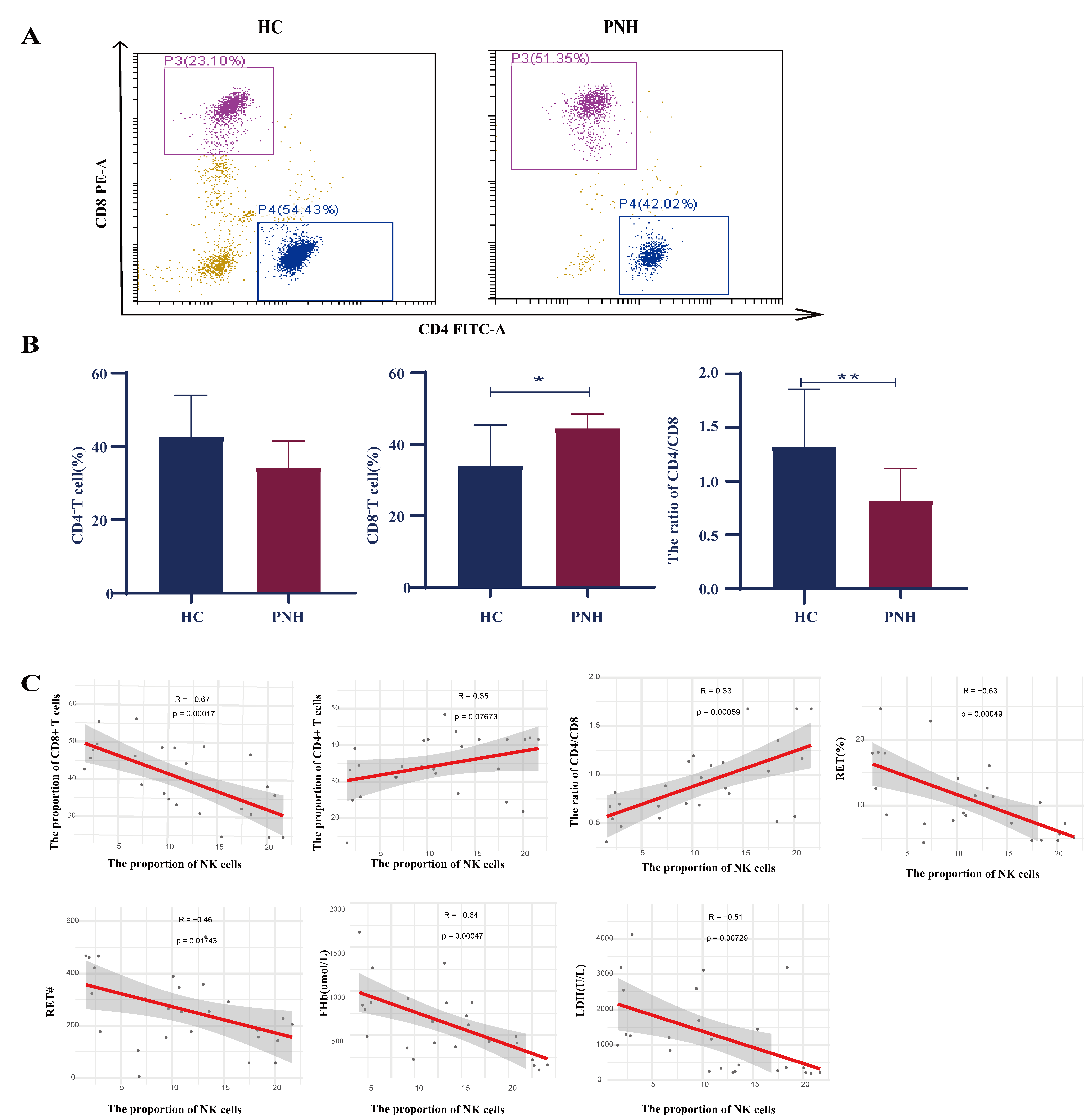

Supplement: Supplementary file 1 — Supporting Information [file CTM2-15-e70542-s003.tif]

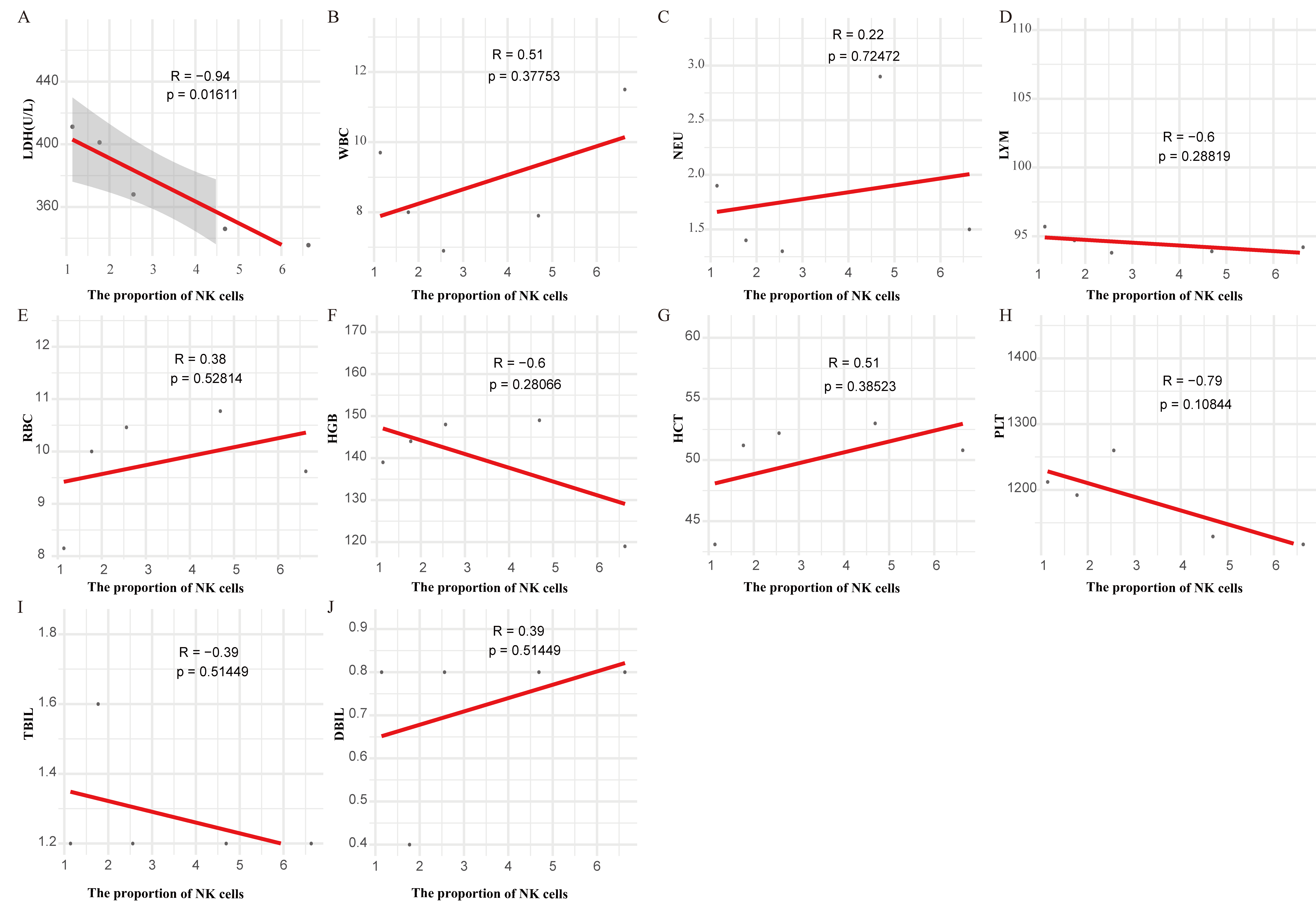

Supplement: Supplementary file 2 — Supporting Information [file CTM2-15-e70542-s005.tif]
