# Supplementary material for: Role of NK cells in immune escape in patients with classical paroxysmal nocturnal haemoglobinuria
Source: Clin Transl Med. 2025 Dec 17;15(12):e70542. doi: 10.1002/ctm2.70542 (PMC12710432; doi:10.1002/ctm2.70542)
Supplement: Supplementary file 3 — Supporting Information [file CTM2-15-e70542-s001.docx]

BM, bone marrow

MDS, myelodysplastic syndrome

ULBP1, UL16 binding protein 1

ULBP2, UL16 binding protein 2

PBMCs, Peripheral Blood Mononuclear Cells

#### *SELL,* [selectin L](https://www.ncbi.nlm.nih.gov/datasets/gene/6402)

#### *FCGR3A,* [Fc gamma receptor IIIa](https://www.ncbi.nlm.nih.gov/datasets/gene/2214)

#### *CXCR4,* [C-X-C motif chemokine receptor 4](https://www.ncbi.nlm.nih.gov/datasets/gene/7852)

#### *PRF1,* [perforin 1](https://www.ncbi.nlm.nih.gov/datasets/gene/5551)

#### *ZEB2，*[zinc finger E-box binding homeobox 2](https://www.ncbi.nlm.nih.gov/datasets/gene/9839)

#### *FCGR3A,* [Fc gamma receptor IIIa](https://www.ncbi.nlm.nih.gov/datasets/gene/2214)

#### *GZMB,* [granzyme B](https://www.ncbi.nlm.nih.gov/datasets/gene/3002)

#### *KLRC2,* [killer cell lectin like receptor C2](https://www.ncbi.nlm.nih.gov/datasets/gene/3822)

#### XCL1, [X-C motif chemokine ligand 1](https://www.ncbi.nlm.nih.gov/datasets/gene/6375)

#### CCL3, [C-C motif chemokine ligand 3](https://www.ncbi.nlm.nih.gov/datasets/gene/6348)

*CCL4,* [C-C motif chemokine ligand 4](https://www.ncbi.nlm.nih.gov/datasets/gene/6348)

*CCL5,* [C-C motif chemokine ligand 5](https://www.ncbi.nlm.nih.gov/datasets/gene/6348)

#### *CCL4L2,* [C-C motif chemokine ligand 4 like 2](https://www.ncbi.nlm.nih.gov/datasets/gene/9560)

#### *C*CL3L1, [C-C motif chemokine ligand 3 like 1](https://www.ncbi.nlm.nih.gov/datasets/gene/9560)

DCs,dendritic cells

#### KLRC3, [killer cell lectin like receptor C3](https://www.ncbi.nlm.nih.gov/datasets/gene/3823)

#### IFNG, [interferon gamma](https://www.ncbi.nlm.nih.gov/datasets/gene/3458)

#### PPP3CA, [protein phosphatase 3 catalytic subunit alpha](https://www.ncbi.nlm.nih.gov/datasets/gene/5530)

#### VAV3, [vav guanine nucleotide exchange factor 3](https://www.ncbi.nlm.nih.gov/datasets/gene/10451)

#### *KLRC1,* [killer cell lectin like receptor C1](https://www.ncbi.nlm.nih.gov/datasets/gene/3821)

#### *IFNAR1,* [interferon alpha and beta receptor subunit 1](https://www.ncbi.nlm.nih.gov/datasets/gene/3454)

#### *HLA-B,* [major histocompatibility complex, class I, B](https://www.ncbi.nlm.nih.gov/datasets/gene/3106)

#### *L*AT, [linker for activation of T cells](https://www.ncbi.nlm.nih.gov/datasets/gene/27040)

#### FYN, [FYN proto-oncogene, Src family tyrosine kinase](https://www.ncbi.nlm.nih.gov/datasets/gene/2534)

#### *DEFA3,*[defensin alpha 3](https://www.ncbi.nlm.nih.gov/datasets/gene/1668)

#### *IL32,* [interleukin 32](https://www.ncbi.nlm.nih.gov/datasets/gene/9235)

#### *PSME2,* [proteasome activator subunit 2](https://www.ncbi.nlm.nih.gov/datasets/gene/5721)

#### *S100A8,* [S100 calcium binding protein A8](https://www.ncbi.nlm.nih.gov/datasets/gene/6279)

#### *S100A9,* [S100 calcium binding protein A9](https://www.ncbi.nlm.nih.gov/datasets/gene/6279)

LDH, lactate dehydrogenase

Th17 (T helper cell 17,Th17)

RP, ribosomal proteins
