# Supplementary material for: Role of NK cells in immune escape in patients with classical paroxysmal nocturnal haemoglobinuria
Source: Clin Transl Med. 2025 Dec 17;15(12):e70542. doi: 10.1002/ctm2.70542 (PMC12710432; doi:10.1002/ctm2.70542)
Supplement: Supplementary file 4 — Supporting Information [file CTM2-15-e70542-s002.docx]

**Supplementary Materials and methods**

**Flow cytometry**

peripheral blood (PB) cells and bone marrow (BM) cells were isolated from fresh sample by density gradient centrifugation, and incubated for 30 min at 4 °C with antibody cocktails in a total volume of 100 μL PBS/5% FBS. To analyze NK cells and their subpopulations, the cells were stained with antibodies targeting the following proteins at the indicated dilutions:CD3(BioLegend;344814,1:20) CD14(BioLegend;367152,1:20),CD19(BioLegend;347544,1:20),CD56(BioLegend;652825,1:20),CD16(BioLegend,335035,1:20),NKG2A(BioLegend;375114,1:20),KIR(R&D;1624104,1:20),NKG2C(R&D;LPZ122212,1:20),CD59(eBioscience;742938,1:100),CD69(BioLegend;310916,1:20),CD45(BioLegend;304048,1:20).

To analyze NK cell function，PB cells were stained with antibodies against CD3(BioLegend;344814,1:20),CD56(BioLegend;362532,1:20),perforin(BioLegend,308106,1:20),CD107a(BioLegend;328608,1:20),CD96(BioLegend;338414,1:20),NKG2A(BioLegend;375114,1:20),NKG2D(BioLegend;320812,1:20),Granzyme(BioLegend;372228,1:20),NKp30(BioLegend;325226,1:20),CD59(BioLegend;304712,1:20).To detect NK cells and their subpopulations in mice,PB cells were stained with antibodies against CD3(BioLegend;317336,1:20),NK1.1(BioLegend;108708,1:20),CD27(BioLegend;124226,1:20),CD11b(BioLegend;101205,1:20).NK cell function:CD3(BioLegend;100204,1:20),NK1.1(BioLegend;108714,1:20),NK1.1(BioLegend;108710,1:20),perforin(BioLegend;154310,1:20),CD107a(BioLegend;121620.1:20),Granzyme(BioLegend;396406,1:20),CD69(BioLegend;104513,1:20),NKG2A(invitrogen;2816196,1:20),NKG2D(BioLegend;115711,1:20),NKp46(BioLegend;137603,1:20),KLRG1(BioLegend;138407,1:20).Flow cytometry was performed on a CytExpert flow cytometer. Dead cells and debris were excluded from the analysis using DAPI staining.Data were analyzed using CytExpert software.

**Isolation and purification of NK cells.**

human peripheral blood was obtained from each patient . CD3-CD56+ cells were freshly purified using NK cell MacS (Miltenyi Biotec, inc.), according to the manufacturer's protocol. Subsequently, cells were detected using a multiparameter flow cytometer ( CytExpert) and analyzed using CytExpert software. The NK cells was collected, the cells were counted and the purity of the cells was measured by flow cytometry. The NK cells were purified by MACS .


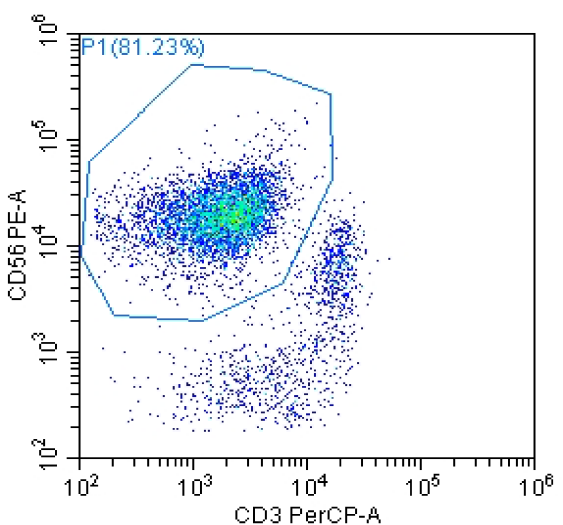


**Figure 1. Purification of NK cells**
