# Supplementary material for: Role of NK cells in immune escape in patients with classical paroxysmal nocturnal haemoglobinuria
Source: Clin Transl Med. 2025 Dec 17;15(12):e70542. doi: 10.1002/ctm2.70542 (PMC12710432; doi:10.1002/ctm2.70542)
Supplement: Supplementary file 5 — Supporting Information [file CTM2-15-e70542-s004.docx]

**SUPPORTING INFORMATION**

Figure S1. **Correlation between the proportion of NK cells in peripheral blood and T cells and clinical indicators of PNH patients was analyzed. A.** T cell subsets were detected by flow cytometry. B. Proportions of CD4^+^T cells and CD8^+^T cells and the ratio of CD4/CD8 were analyzed statistically between classical PNH and HC. C. Correlation analysis between the proportion of NK cells in peripheral blood and clinical parameters in patients with classical PNH (**P*<0.05;***P*<0.01;****P*<0.001).


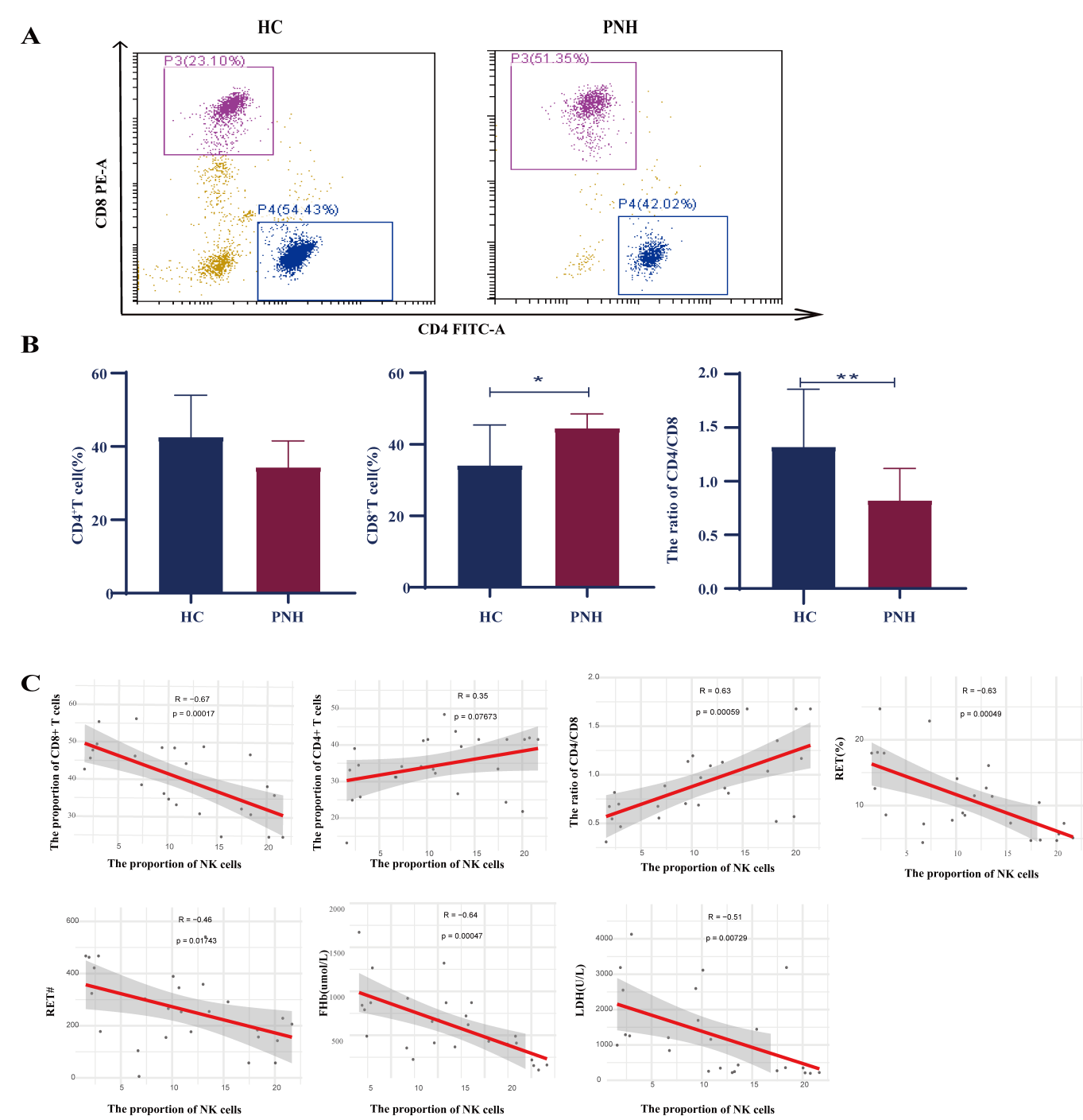


Figure S2. **Correlation analysis between the proportion of NK cells in peripheral blood and clinical parameters in CKO homozygous mice**


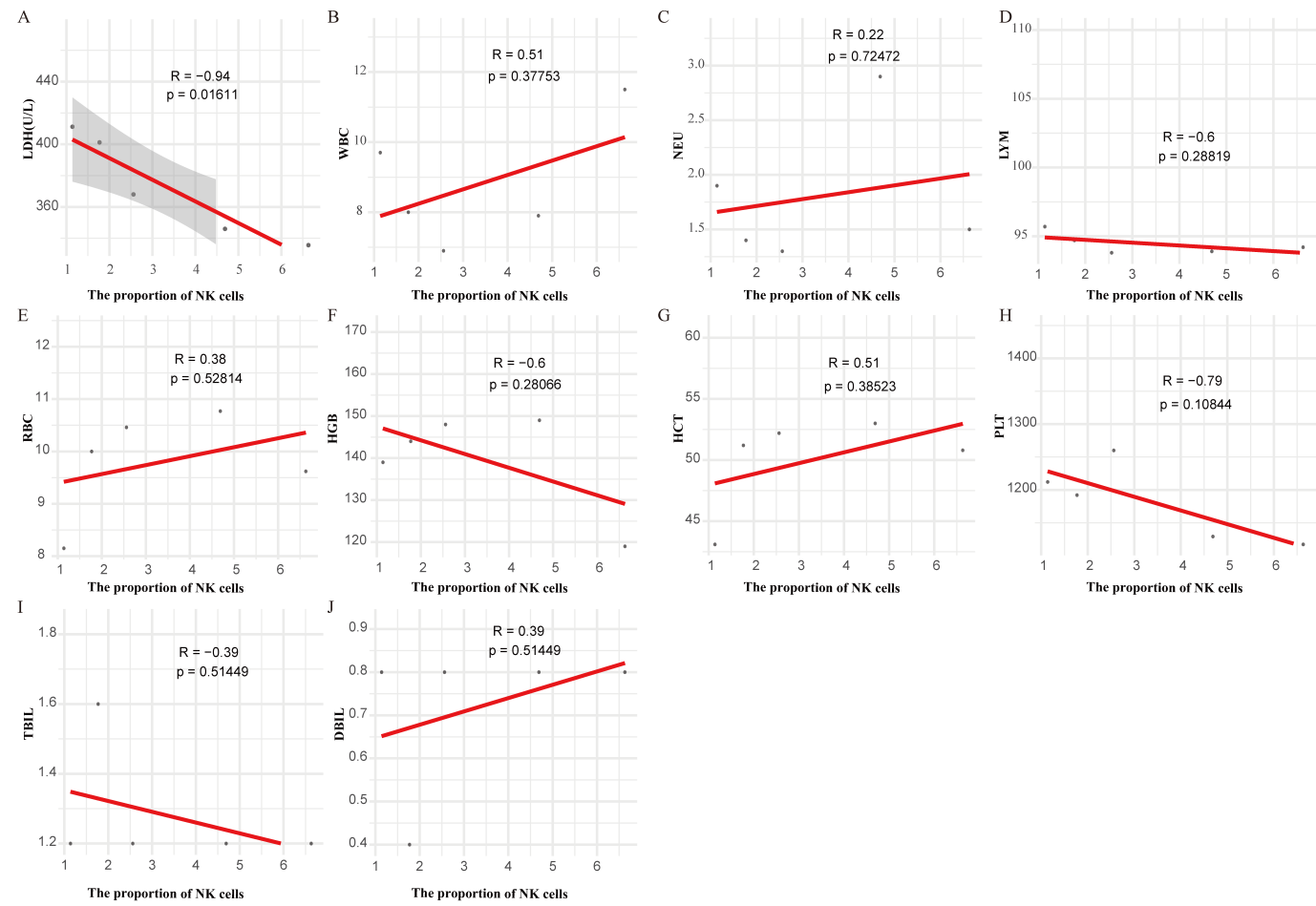


**Table S1 Baseline Characteristics of PNH Patients**

| Clinical indicators | Mean (SD)/Median (P25, P75) | **Reference Values** |
| --- | --- | --- |
| Male/Female (N) | 12/14 |  |
| Age (range) | 12-58 |  |
| Time since diagnosis (years) | 6.35±4.39 |  |
| History of thrombosis (N) | 3/26 |  |
| Complete Blood Count |  |  |
| RBC (×10^12/L) | 2.60±0.78 | 4.30－5.80 |
| HB (g/L) | 83.08±24.06 | 130－175 |
| WBC (×10^9/L) | 3.58 (2.26, 5.47) | 3.50－9.50 |
| PLT (×10^9/L) | 143.54±68.91 | 125－350 |
| RET (%) | 11.05±5.69 | 0.50－1.50 |
| RET (×10^9/L) | 261.26±141.78 | 24.00－84.00 |
| Biochemical indicators |  |  |
| LDH (U/L) | 1283.03±1161.90 | 120－250 |
| TBIL (μmol/L) | 37.65 (28.95, 57.70) | ≤26 |
| IBIL (μmol/L) | 30.05 (22.08, 52.63) | 1.7－12 |
| Coagulation function |  |  |
| Fibrinogen (g/L) | 2.71±1.03 | 1.80－4.00 |
| D-Dimer (ng/ml) | 296.51 (146.0, 782.0) | 0－500 |
| PNH clones |  |  |
| CD59-Erythrocytes (%) | 54.99±28.52 |  |
| CD59-Neutrophils | 82.85±13.59 |  |
| CD14-Flaer- (%) | 88.33±8.77 |  |
| CD24-Flaer- (%) | 88.93±10.15 |  |
| Free hemoglobin (mg/L) | 627.78±385.70 | <40 |

Note: RBC, erythrocytes; HB, hemoglobin;WBC, white blood cells; PLT, platelets; RET, reticulocyte; LDH, lactate dehydrogenase; TBIL, total bilirubin; IBIL, indirect bilirubin; PNH, Paroxysmal nocturnal hemoglobinuria

**Table S2. Proportion NK cells detected by scRNA-seq**

| Terms (%) | Control  (n=4) | CD59^+^NK cells  (n=4) | CD59^-^NK cells  (n=4) |
| --- | --- | --- | --- |
|  | Median (P25, P75) | | |
| Active NK | 4.96 (1.64, 5.64) | 31.87 (6.85, 82.33)^c^ | 0.32 (0, 2.0)^a^ |
| Mature NK | 49.84 (17.88, 68.16) | 2.33 (0.55, 7.08)^b^ | 14.43 (0.21, 63.37) |
| Adaptive NK | 8.94 (3.76, 18.80) | 44.85 (11.20, 56, 36) | 1.85 (0.16, 16.80) |
| Transitional NK | 3.24 (0.76, 7.76) | 8.85 (2.65, 30.24)^c^ | 0 (0, 1.66) |
| Inflamed NK | 15.26 (7.54, 57.91) | 0.64 (0.14, 5.52)^bc^ | 13.81 (7.24, 22.33) |
| Terminally NK | 2.54 (0.63, 3.97) | 0.20 (0.02, 0.37)^c^ | 28.07 (1.92, 61.28) |
| CD56^bright^ NK | 6.39 (3.94, 10.43) | 0.89 (0.40, 6.67)^c^ | 15.33 (13.87, 35.37)^a^ |

“a”CD59^-^NK group is statistically significant compared with the control

“b”CD59^+^NK group is statistically significant compared with the control

“c”CD59^+^NK group is statistically significant compared with the CD59^-^NK group

**Table S3. Proportion of NK cells in PB detected via FCM**

| Terms (%) | PNH (n=26) | HC (n=27) | t/Z value | P value |
| --- | --- | --- | --- | --- |
|  | Median (P25, P75)/Mean±SD | |  |  |
| NK cells | 11.13±6.55 | 17.00±9.53 | -2.61 | **0.01** |
| CD56^bright^ NK | 8.80±5.55 | 5.32±3.87 | 2.65 | **0.01** |
| CD56^dim^NK | 71.51 (51.40, 86.13) | 88.53 (81.97, 94.68) | -3.50 | **<0.001** |
| Mature NK | 50.92±22.45 | 60.26±18.28 | -1.67 | 0.10 |
| Terminal NK | 12.91 (8.36, 21.07) | 8.99 (5.21, 13.22) | -1.95 | 0.05 |
| Adaptive NK | 21.08 (5.15, 39.05) | 8.01 (2.35, 18.81) | -2.23 | **0.03** |
| Active NK | 16.57 (6.00, 30.87) | 6.07 (2.68, 10.98) | -3.10 | **0.002** |

**Table S4. Proportion of CD59^-^ and CD59^+^NK cells in PB detected via FCM**

| Terms (%) | HC (n=27) | CD59^-^NK (n=26) | CD59^+^NK (n=26) |
| --- | --- | --- | --- |
|  | Median (P25, P75)/Mean±SD | | |
| NK cells | 17.00±9.53 | 3.95±4.31^a^ | 10.29±6.47^bc^ |
| CD56^bright^ NK | 5.32±3.87 | 10.20 (4.45, 12.40)^a^ | 7.48 (3.60, 14.38) |
| CD56^dim^NK | 88.53 (81.97, 94.68) | 75.10 (50.88, 87.06)^a^ | 81.85 (60.96, 90.04)^b^ |
| Mature NK | 60.26±18.28 | 47.50±23.34^a^ | 57.55±22.64 |
| Terminal NK | 8.99 (5.21, 13.22) | 10.03 (5.98, 18.54) | 8.26 (3.23, 14.74) |
| Adaptive NK | 8.01 (2.35, 18.81) | 22.44 (5.97, 32.11) | 29.46 (6.0, 47.10)^b^ |
| Active NK | 6.07 (2.68, 10.98) | 5.30 (1.42, 12.78) | 19.62 (7.54, 44.10)^bc^ |

“a” CD59^-^NK group is statistically significant compared with the HC group

“b”CD59^+^NK group is statistically significant compared with the HC group

“c”CD59^+^NK group is statistically significant compared with the CD59^-^NK group

**Table S5. Proportion of NK cells in BM detected via FCM**

| Terms (%) | PNH (n=14) | HC (n=20) | t/Z value | P value |
| --- | --- | --- | --- | --- |
|  | Median (P25, P75)/Mean±SD | |  |  |
| NK cells | 17.79 (12.35, 27.35) | 13.11 (6.45, 22.10) | -1.33 | 0.18 |
| CD56^bright^ NK | 10.07 (5.87, 15.52) | 5.48 (3.58, 9.05) | -2.42 | **0.02** |
| CD56^dim^NK | 70.58 (62.46, 78.29) | 89.22 (79.72, 94.55) | -3.57 | **<0.001** |
| Mature NK | 53.19±16.60 | 65.89±17.60 | -2.12 | **0.04** |
| Terminal NK | 13.05±7.44 | 11.61±6.29 | 0.613 | 0.54 |
| Adaptive NK | 29.58 (2.62, 37.75) | 11.12 (4.48, 15.04) | -1.645 | 0.10 |
| Active NK | 12.25±6.44 | 7.66±4.38 | 2.479 | **0.02** |

**Table S6. Proportion of CD59^-^ and CD59^+^NK cells in BM detected via FCM**

| Terms (%) | HC (n=20) | CD59-NK (n=14) | CD59+NK (n=14) |
| --- | --- | --- | --- |
|  | Median (P25, P75)/Mean±SD | | |
| NK cells | 13.11 (6.45, 22.10) | 2.25 (1.28, 4.41)a | 13.04 (8.91, 22.22)c |
| CD56^bright^ NK | 6.16±3.31 | 15.15±8.13^a^ | 8.94±4.74^c^ |
| CD56^dim^NK | 86.26±9.83 | 64.07±17.69^a^ | 71.48±16.25^b^ |
| Mature NK | 65.89±17.60 | 45.84±17.97^a^ | 52.77±22.80 |
| Terminal NK | 10.72 (6.73, 14.67) | 15.56 (4.79, 34.17) | 6.28 (3.26, 38.16) |
| Adaptive NK | 11.12 (4.48，15.04) | 16.85 (10.73, 20.01) | 21.37 (11.75, 36.74) |
| Active NK | 7.12 (3.88, 11.70) | 10.11 (6.32, 12.32) | 14.02 (11.79, 27.44)^bc^ |

“a” CD59^-^NK group is statistically significant compared with the HC group

“b”CD59^+^NK group is statistically significant compared with the HC group

“c”CD59^+^NK group is statistically significant compared with the CD59^-^NK group

**Table S7. Functions of NK cells in PB detected via FCM**

| Terms (%) | PNH (n=26) | HC (n=27) | Z value | P value |
| --- | --- | --- | --- | --- |
|  | Median (P25, P75) | |  |  |
| Perforin | 65.29 (57.93, 71.95) | 78.47 (64.56, 85.00) | -2.49 | **0.013** |
| NKG2D | 80.25 (68.06, 86.86) | 87.30 (74.14, 92.36) | -2.153 | **0.031** |
| GZMB | 71.76 (59.73, 76.97) | 75.26 (64.07, 80.61) | -1.201 | 0.230 |
| CD107a | 19.79 (14.65, 23.04) | 25.76 (18.87, 41.88) | -2.793 | **0.005** |
| CD96 | 33.58 (26.55, 41.40) | 24.43 (16.49, 31.17) | -3.203 | **0.001** |
| NKG2A | 26.89 (12.42, 45.95) | 34.19 (25.68, 42.68) | -1.085 | 0.278 |
| NKp30 | 28.22 (21.85, 33.99) | 33.31 (27.84, 40.62) | -2.028 | **0.043** |

**Table S8. Function of CD59^-^ and CD59^+^NK cells in PB detected via FCM**

| Terms (%) | HC (n=27) | CD59^-^NK (n=26) | CD59^+^NK (n=26) |
| --- | --- | --- | --- |
|  | Median (P25, P75) | | |
| Perforin | 78.47 (64.56, 85.00) | 61.37 (36.61, 78.07)^a^ | 83.02 (73.20, 91.81)^c^ |
| NKG2D | 87.30 (74.14, 92.36) | 79.94 (58.40, 91.73) | 87.28 (66.64, 94.62) |
| GZMB | 75.26 (64.07, 80.61) | 64.27 (42.24, 84.78) | 79.74 (58.31, 91.83)^c^ |
| CD107a | 25.76 (18.87, 41.88) | 18.01 (10.98, 28.22)^a^ | 23.66 (18.04, 31.27) |
| CD96 | 24.43 (16.49, 31.17) | 19.74 (8.82, 28.80) | 35.74 (22.45, 47.00)^bc^ |
| NKG2A | 34.19 (25.68, 42.68) | 29.48 (17.54, 52.24) | 22.75 (18.13, 37.47)^b^ |
| NKp30 | 33.31 (27.84, 40.62) | 23.41 (15.10, 42.80) | 29.27 (18.64, 41.95) |

“a” CD59^-^NK group is statistically significant compared with the HC group

“b”CD59^+^NK group is statistically significant compared with the HC group

“c”CD59^+^NK group is statistically significant compared with the CD59^-^NK group

**Table S9. Proportion of NK cells of mice in PB detected via FCM**

| Terms (%) | Flox mice (n=5) | CKO homozygous mice (n=5) | Z value | P value |
| --- | --- | --- | --- | --- |
|  | Median (P25, P75) | |  |  |
| NK cell | 8.97 (8.30, 11.16) | 3.56 (1.46, 5.66) | -2.61 | **0.01** |
| Immature NK | 7.61 (3.88, 10.43) | 8.64 (3.10, 11.47) | -0.31 | 0.75 |
| Early mature NK | 8.75 (7.32, 11.24) | 4.10 (1.91, 6.25) | -2.40 | **0.02** |
| Mature NK | 15.25 (13.70, 19.56) | 8.61 (6.98, 9.79) | -2.61 | **0.01** |
| Late mature NK | 68.34 (63.47, 70.50) | 80.37 (75.95, 84.29) | -2.61 | **0.01** |

**Table S10. Function of NK cells of mice in PB detected via FCM**

| Terms (%) | Flox mice (n=5) | CKO homozygous mice (n=5) | Z value | P value |
| --- | --- | --- | --- | --- |
|  | Median (P25, P75) | |  |  |
| perforin | 55.15 (49.12, 69.90) | 29.76 (25.33, 42.50) | -2.40 | **0.02** |
| Granzyme B | 28.80 (25.30, 37.72) | 24.13 (19.79, 33.94) | -0.73 | 0.47 |
| CD107a | 45.15 (38.59, 58.05) | 22.11 (17.14, 28.65) | -2.61 | **0.01** |
| NKG2D | 44.56 (33.07, 47.80) | 39.39 (31.61, 41.38) | -1.15 | 0.25 |
| NKp46 | 25.16 (17.41, 33.49) | 20.87 (12.40, 28.26) | -0.73 | 0.47 |
| KLRG1 | 32.22 (23.53, 41.15) | 52.33 (40.79, 59.08) | -2.19 | **0.03** |
| NKG2A | 29.50 (25.13, 31.09) | 47.37 (41.50, 61.21) | -1.37 | 0.17 |
| CD69 | 36.59 (28.97, 46.82) | 21.05 (15.56, 24.67) | -2.40 | **0.02** |
